# Antioxidant strategies of four native tree species across seasons in urban and peri-urban Atlantic Forest remnants

**DOI:** 10.1007/s10661-026-15732-0

**Published:** 2026-07-23

**Authors:** Bruno Ruiz Brandão da Costa, Fernanda Anselmo-Moreira, Allanis Catharina Pimenta Corrêa, Adriana dos Santos Lopes, Hugo Humberto de Araújo, Graciele Daiane Diniz Soares, Júlia Duarte Mendes, Adalgiza Fornaro, Agnès Borbon, Silvia Ribeiro de Souza, Luzimar Campos da Silva, Cláudia Maria Furlan

**Affiliations:** 1 Departamento de Botânica, Instituto de Biociências, Universidade de São Paulo, Rua Do Matão, 277, São Paulo, SP 05508-090 Brazil; 2 Núcleo de Uso Sustentável de Recursos Naturais, Unidade Jardim Botânico, Instituto de Pesquisas Ambientais, São Paulo, SP 04301-002 Brazil; 3 Departamento de Biologia Vegetal, Universidade Federal de Viçosa, Viçosa, MG 36570-900 Brazil; 4 Departamento de Ciências Atmosféricas, Instituto de Astronomia, Geofísica E Ciências Atmosféricas, Universidade de São Paulo, São Paulo, SP 05508-090 Brazil; 5 Université Clermont Auvergne, Laboratoire de Météorologie Physique, LaMP/CNRS, 6016 Clermont-Ferrand, France

**Keywords:** Leaf redox homeostasis, Ascorbate–glutathione cycle, Abiotic stress, Plant anatomy, Tropospheric ozone

## Abstract

Urban Atlantic Forest remnants are exposed to atmospheric oxidants, particularly tropospheric ozone (O₃), and seasonal environmental variability, yet the biochemical basis of tree resilience to these combined pressures remains poorly resolved. We investigated antioxidant strategies in *Alchornea sidifolia* (AS), *Casearia sylvestris* (CS), *Guarea macrophylla* (GM), and *Machaerium nyctitans* (MN), comparing an urban and a peri-urban site across dry and rainy seasons in São Paulo, Brazil. We quantified ascorbate (AsA) and glutathione (GSH) redox pools, antioxidant enzymes (superoxide dismutase, SOD; catalase, CAT; and ascorbate peroxidase, APX), total phenolic content (TPC), and malondialdehyde (MDA), and integrated these biochemical data with leaf anatomical analyses and cell death assays. Biochemical profiles were structured by species and, to a lesser extent, by season (PERMANOVA: species *R*^2^ = 0.292, *p* = 0.001; season *R*^2^ = 0.037, *p* = 0.010; site *R*^2^ = 0.003, *p* = 0.956). Compact-mesophyll species (AS and CS) showed greater redox stability, but with distinct outcomes. AS combined high constitutive SOD, large AsA pools, and the highest TPC, yet displayed epidermal/mesophyll damage and increased cell death under higher oxidative pressure. CS maintained stable AsA-GSH partitioning, high APX capacity, and minimal anatomical damage across conditions. GM and MN, porous-mesophyll species, did not show coordinated antioxidant compensation and showed greater anatomical damage and cell death. Across species, damage increased in the rainy season, when water availability, temperature, irradiance, and O₃ exposure were higher. Site-related differences were expressed mainly as tissue-level damage and antioxidant pool depletion rather than as coordinated enzymatic induction. Thus, resilience to urban O₃-related oxidative stress and seasonal environmental variability was associated with the integration of structural traits and constitutive redox buffering rather than with coordinated inducible antioxidant responses. These patterns support the use of combined redox and tissue-integrity markers to identify complementary native species for biomonitoring oxidative-stress responses in Atlantic Forest remnants.

**Graphical abstract:**

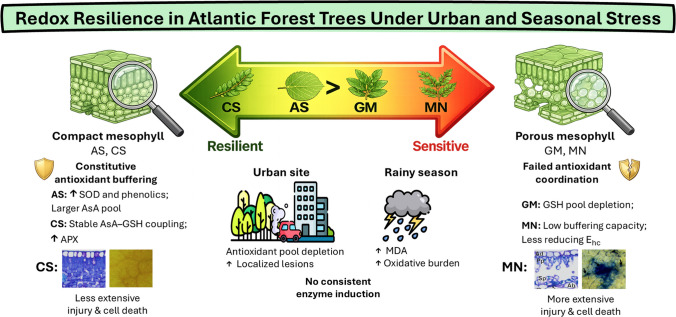

**Supplementary Information:**

The online version contains supplementary material available at 10.1007/s10661-026-15732-0.

## Introduction

The Brazilian Atlantic Forest is among the world’s most species-rich biomes, yet also one of the most threatened owing to fragmentation and increasing exposure to urban pollution and climate variability (Amaral et al., 2025). These pressures can impair plants by disrupting redox homeostasis, creating an imbalance between reactive oxygen species (ROS) production and detoxification (Mittler et al., 2022). Although ROS serve as essential signaling molecules under basal conditions, their excess overwhelms detoxification and repair systems, causing oxidative damage to lipids, proteins, and nucleic acids, loss of membrane integrity, and anatomical and physiological dysfunction (Considine & Foyer, 2021).

Plants maintain cellular integrity through a multilayered antioxidant system. Core enzymes such as superoxide dismutase (SOD), catalase (CAT), and ascorbate peroxidase (APX) limit ROS accumulation, while low-molecular-weight antioxidants, particularly ascorbate (AsA) and glutathione (GSH), maintain redox buffering through regeneration (Dumanović et al., 2021; Foyer & Kunert, 2024). Secondary metabolites, including tocopherols, carotenoids, and polyphenols, also contribute to antioxidant defense. In this context, redox biomarkers and oxidative damage indicators, such as lipid peroxidation indexed by malondialdehyde (MDA), help assess in situ oxidative stress responses and potential tolerance (Esposito et al., 2018; Noctor et al., 2016).

Although air pollution and climate variability are well-established drivers of oxidative stress, mechanistic field evidence for native Atlantic Forest trees remains limited. Early studies identified native tree species for passive pollution biomonitoring and screened morphological, chemical, and biochemical biomarkers to assess bioindicator potential (Domingos et al., 2015). They also showed that antioxidant responses vary with species identity and environmental context: metabolic regulation shifts between rainy-season photooxidative conditions and dry-season drought pressure (Aguiar-Silva et al., 2016), and pioneer species tend to maintain stronger defenses than non-pioneers (Esposito et al., 2018). Anatomical analyses complement biochemical approaches by revealing tissue-level evidence of tolerance and sensitivity. Necrosis, cell collapse, protoplast retraction, and altered cell shape are linked to redox imbalance and have been reported in both controlled exposure studies and native vegetation fragments in urban environments (Fernandes & Moura, 2021; Moura et al., 2018; Silva et al., 2023), while leaf anatomical architecture is itself related to responses to environmental stressors and seasonality (de Araújo et al., 2025; Oksanen & Kontunen-Soppela, 2021). Together, these studies establish redox and anatomical traits as useful in situ indicators of environmental stress, but the available evidence remains taxonomically and geographically narrow, limiting broader inference across abundant taxa and urban forest settings.

This gap is especially relevant in the Metropolitan Area of São Paulo (MASP), where Atlantic Forest remnants are chronically exposed to urban air pollutants. In this urban matrix, recent research compared two ecologically contrasting sites: the urban-exposed Biosciences Institute Forest Reserve (Matão-IAG) and the peri-urban Morro Grande Forest Reserve (RMG) (de Araújo et al., 2025). The study assessed four native tree species, *Alchornea sidifolia* (AS), *Casearia sylvestris* (CS), *Guarea macrophylla* (GM), and *Machaerium nyctitans* (MN), and classified AS and CS as more tolerant, and GM and MN as more sensitive, to atmospheric pollution, particularly tropospheric ozone (O₃), based on visual, morphoanatomical, physiological, and volatile-emission markers. In that study, tolerance was associated mainly with lower visual and structural leaf damage and compact mesophyll architecture, whereas sensitivity was associated with greater tissue damage and porous mesophyll. However, although leaf architecture was linked to pollution sensitivity, the biochemical basis of these responses remains unresolved, and the role of climatic seasonality requires further investigation.

Therefore, this study characterized the oxidative stress responses of AS, CS, GM, and MN by comparing redox status across two contrasting seasons (dry and rainy) and sites (urban Matão-IAG and peri-urban disturbed RMG). We hypothesized that (i) species differ in redox regulatory capacity, with the previously identified pollution/O₃-tolerant species (AS and CS), characterized by compact mesophyll, showing greater redox stability than the pollution/O₃-sensitive species (GM and MN), which have porous mesophyll; (ii) the dry season increases oxidative burden and shifts leaf redox status toward oxidation; and (iii) the more urbanized site is associated with greater oxidative challenge and increased demand for antioxidant buffering capacity.

To test these hypotheses, we quantified lipid peroxidation (MDA), antioxidant enzyme activities (SOD, CAT, and APX), the redox status of the ascorbate and glutathione pools (AsA and GSH), and total phenolic content (TPC), and integrated these data with microscopy-based evidence of leaf anatomical damage and cell death. This integrative approach helps interpret species sensitivity and resilience to urban oxidant exposure and seasonal abiotic variability in urban Atlantic Forest remnants and informs conservation and biomonitoring under increasing urban pressure.

## Material and methods

### Study areas 

The study was conducted in two secondary Atlantic Forest remnants within the MASP that differ in anthropogenic influence. Morro Grande Forest Reserve (RMG) (23°39′–23°48′S, 47°01′–46°55′W), located about 34 km west of central São Paulo, is a preserved peri-urban forest with limited human disturbance. It comprises Dense Ombrophilous Montane Forest in advanced successional stages and has a temperate oceanic climate (Cfb) according to the Köppen classification (Metzger et al., 2006). The Biosciences Institute Forest Reserve (Matão-IAG) (23°33′–23°34′S, 46°43′W) is located on the University of São Paulo (USP) campus, within a densely urbanized area, and comprises vegetation with species typical of both Dense Ombrophilous Forest and Semideciduous Seasonal Forest. The site has a warm temperate climate (Cwa) (Dislich & Pivello, 2002) and is affected by vehicular emissions and urban heat-island effects. Detailed site descriptions and maps are available in previous studies (Borbon et al., 2026; de Araújo et al., 2025).

### Sampling design and environmental data

Sampling was conducted during the dry season (winter, August–September 2023) and the rainy season (summer, January–March 2024) to capture seasonal variation. Daily temperature and precipitation were obtained from the on-site tower at RMG and the IAG-USP station at Matão-IAG. Photosynthetically active radiation (PAR) and ultraviolet (UV) irradiance were retrieved from the NASA POWER database v2.5.25 (https://power.larc.nasa.gov/). Hourly O₃ concentrations were obtained from the nearest monitoring stations, Carapicuíba for RMG and Cidade Universitária (USP-IPEN) for Matão-IAG, and summarized as AOT40 (Accumulated ozone exposure over a threshold of 40 ppb) and the sum of all hourly values (SUM00), following de Araújo et al. (2025).

### Plant species selection

We selected four native tree species from distinct botanical families based on their high abundance and structural dominance across both study sites (Borbon et al., 2026): *Alchornea sidifolia* Müll.Arg. (Euphorbiaceae), *Casearia sylvestris* Sw. (Salicaceae), *Guarea macrophylla* Vahl (Meliaceae), and *Machaerium nyctitans* (Vell.) Benth. (Fabaceae).

### Biochemical analyses

#### Sample collection

Sun-exposed branches were harvested, and healthy leaves were collected, weighed, immediately frozen on dry ice, transported to the laboratory, and stored at − 80 °C until analysis. Six biological replicates per species were collected for each site × season combination (*n* = 6). All absorbance measurements were obtained using a Synergy H1 microplate reader.

#### Non-enzymatic antioxidants: Ascorbate (AsA) and glutathione (GSH)

Ascorbate and glutathione pools were quantified using a method adapted from Sala-Carvalho et al. (2022), with full extraction and chromatographic details provided in Supplementary Methods. For ascorbate, the reduced form was reported as ascorbate (AsA), the oxidized form as dehydroascorbate (DHA), and the total pool as total ascorbate (TotalAsA), calculated as TotalAsA = AsA + DHA. For glutathione, the reduced form was reported as reduced glutathione (GSH), the oxidized form as glutathione disulfide (GSSG), and the total pool as total glutathione (TotalGSH), calculated as TotalGSH = GSH + GSSG. Concentrations were expressed as µmol g⁻^1^ FM, where FM denotes fresh mass. Ascorbate redox status was expressed as the ascorbate reduction ratio (RatioAsA), calculated as RatioAsA = AsA/TotalAsA: this ratio represents the fraction of the total ascorbate pool present in the reduced form. Glutathione redox status was expressed as the half-cell reduction potential (E_hc_), calculated from [GSH] and [GSSG] in mol L⁻^1^ using the Nernst equation, assuming E°′ = − 240 mV at 25 °C and pH 7.0 (Bela et al., 2018; Schafer & Buettner, 2001):

1 $${\mathrm{E}}_{\mathrm{h}\mathrm{c}} (\mathrm{m}\mathrm{V})\hspace{0.17em}=\hspace{0.17em}\hspace{0.17em}-\hspace{0.17em}240\hspace{0.17em}-\hspace{0.17em}(59.1/2) \cdot {\mathrm{l}\mathrm{o}\mathrm{g}}_{10}(([\mathrm{G}\mathrm{S}\mathrm{H}{]}^{2})/[\mathrm{G}\mathrm{S}\mathrm{S}\mathrm{G}])$$

#### Total phenolic content (TPC)

TPC was determined by the Folin–Ciocalteu method following Furlan et al. (2015), with procedural details provided in Supplementary Methods. Phenolics were extracted from frozen ground leaf tissue (50 mg) with 80% methanol, and absorbance was measured at 760 nm after reaction with Folin–Ciocalteu reagent. Quantification was based on a gallic acid standard curve, and TPC was expressed as mg g⁻^1^ FM.

#### Antioxidant enzyme activities

Enzyme extraction and activity assays followed Lopes and Furlan (2025), with full assay conditions provided in Supplementary Methods. Catalase (CAT) activity was determined from the decrease in absorbance at 240 nm due to H₂O₂ decomposition and expressed as µmol H₂O₂ min⁻^1^ g⁻^1^ FM. Ascorbate peroxidase (APX) activity was determined from the H₂O₂-dependent oxidation of AsA at 290 nm and expressed as µmol AsA oxidized min⁻^1^ g⁻^1^ FM. Superoxide dismutase (SOD) activity was determined from inhibition of nitro blue tetrazolium photoreduction at 560 nm. One unit of SOD activity was defined as the amount of enzyme causing 50% inhibition, and activity was expressed as U g⁻^1^ FM.

#### Malondialdehyde (MDA)

Lipid peroxidation was estimated as malondialdehyde (MDA) equivalents using the thiobarbituric acid-reactive substances assay of Hodges et al. (1999), with full procedural and correction details provided in Supplementary Methods. Frozen ground leaf tissue (200 mg) was extracted in 80% ethanol, reacted with thiobarbituric acid, and absorbance was measured at 440, 532, and 600 nm. MDA equivalents were calculated from a standard curve, corrected for extraction volume, and expressed as nmol g⁻^1^ FM.

### Leaf tissue integrity

For each site × season combination, fully expanded leaves (*n* = 3) were collected from the third node below the apex for anatomical and cell death analyses. Sampling was performed on a subset of the same trees used for biochemical analyses in the four study species.

#### Leaf anatomical damage

Leaf fragments were fixed in neutral buffered formalin (Kraus & Arduin, 1997), dehydrated through an ethanol series, and embedded in Historesin (Leica Instruments, Heidelberg, Germany). Transverse sections (5 µm) were obtained using a Leica RM 2155 rotary microtome (Leica Microsystems GmbH, Wetzlar, Germany). Sections were stained with 0.05% toluidine blue at pH 4.7 (O’Brien & McCully, 1981) and mounted on permanent slides with Permount (Thermo Fisher Scientific). Slides were examined and documented using an AX70TRF photomicroscope (Olympus Optical, Tokyo, Japan) coupled to an image capture system (Axio Vision Release 4.8.1, Carl Zeiss Vision GmbH, Germany).

#### Cell death assay

Leaf discs were excised and incubated in 0.25% Evans Blue solution for approximately 2 h (Romero-Puertas et al., 2004). Samples were then clarified in 95% ethanol at 65 °C for 24 h. Intense blue staining was interpreted as evidence of increased membrane permeability and cell death. Observations and image acquisition were performed using a stereomicroscope (Olympus SZXF).

### Statistical analysis

All analyses were performed in R v4.5.1. Biochemical variables were standardized before multivariate analyses. Multivariate patterns were explored using Principal Component Analysis (PCA), with temperature, precipitation, AOT40, photosynthetically active radiation (PAR), and UV fitted as supplementary vectors. Group differences were tested by Permutational Multivariate Analysis of Variance (PERMANOVA) using Euclidean distances and 999 permutations. Redundancy Analysis (RDA) was used to assess the contribution of environmental predictors and spatiotemporal factors to biochemical variation, including models for environmental variables, Site, Season, Site × Season, and partial effects of Site and Season. Model and term significance were assessed by permutation-based ANOVA with 999 permutations. Pearson correlation matrices were used to examine associations between individual biochemical and environmental variables.

Species-level responses were tested using regression models for each oxidative-stress and redox-related variable, with Site, Season, and Site × Season as predictors. Linear models were used when residual normality and variance homogeneity were met; otherwise, Gamma generalized linear models with log-link functions were fitted for strictly positive variables. Candidate models included Site × Season, Site + Season, Site only, Season only, and an intercept-only model, and were ranked using Akaike’s Information Criterion and AIC weights. Estimated marginal means (EMMs) were compared using Sidak-adjusted pairwise tests. Interspecific differences in RatioAsA, E_hc_, and TPC were assessed with full-factorial Species × Site × Season models followed by Tukey-adjusted comparisons.

## Results

### Environmental conditions

Environmental conditions followed the expected seasonal pattern for São Paulo, with warmer and wetter conditions in the rainy season and cooler, drier conditions in the dry season (Borbon et al., 2026; dos Santos et al., 2022). In situ data confirmed this pattern and showed that Matão-IAG was consistently warmer than RMG (Table 1). Rainfall increased from 74 to 426 mm at RMG and from 69 to 524 mm at Matão-IAG between the dry and rainy seasons, while mean temperature rose from 15.1 to 22.5 °C and from 18.2 to 23.8 °C, respectively.

**Table 1:** Summary of environmental conditions and ozone (O₃) exposure at two sites during the dry (June–August 2023) and rainy (December 2023–February 2024) seasons, calculated for the three months preceding the final sampling date

Site	Season	Precipitation (mm, cumulative)	Temperature (°C, mean [min–max])	Mean PAR (MJ m⁻^2^ day⁻^1^)	Mean UV	SUM00 (ppm h)	AOT40 (ppb h)
RMG	Dry	74	15.1 (7.3–24.8)	5.99	0.95	29	773
RMG	Rainy	426	22.5 (15.1–33.9)	9.58	2.36	44	3402
Matão-IAG	Dry	69	18.2 (9.8–29.6)	5.99	0.95	38	4021
Matão-IAG	Rainy	524	23.8 (16.6–34.4)	9.58	2.36	53	8498

*RMG* Morro Grande Forest Reserve; *Matão-IAG* Biosciences Institute Forest Reserve; *PAR* photosynthetically active radiation; *UV* ultraviolet radiation; O₃, ozone; *SUM00* sum of all hourly ozone concentrations; *AOT40* accumulated ozone exposure over a threshold of 40 ppb, where ppb denotes parts per billion. Temperature and precipitation data were obtained from the on-site tower at the Morro Grande Forest Reserve (RMG) and from the Institute of Astronomy, Geophysics and Atmospheric Sciences of the University of São Paulo station (IAG-USP) at the Biosciences Institute Forest Reserve (Matão-IAG). Precipitation values represent cumulative totals, and temperature values represent daily means, with minimum and maximum values in parentheses. Hourly ozone (O₃) concentrations were obtained from the nearest air quality monitoring stations: Carapicuíba for RMG and Cidade Universitária, University of São Paulo–Nuclear and Energy Research Institute (USP-IPEN), for Matão-IAG. These data were summarized as the sum of all hourly ozone concentrations (SUM00) and accumulated ozone exposure over a threshold of 40 ppb (AOT40). Monthly mean photosynthetically active radiation (PAR) and monthly mean surface ultraviolet (UV) index were retrieved from the NASA POWER database. The UV index is a dimensionless metric defined as 40 times the biologically effective ultraviolet irradiance, expressed in watts per square metre (W m⁻^2^)

O₃ exposure was higher at Matão-IAG in both seasons and increased during the rainy season at both sites. AOT40 rose from 773 to 3402 ppb h at RMG and from 4021 to 8498 ppb h at Matão-IAG, exceeding the 3000 ppb h vegetation-risk threshold during the rainy season at both sites (Cakaj et al., 2023). PAR and UV varied mainly with season, increasing from 5.99 to 9.58 MJ m⁻^2^ day⁻^1^ and from 0.95 to 2.36, respectively. These conditions indicate that the rainy season combined higher water availability with greater irradiance and O₃ exposure, especially at the urban site.

### Oxidative stress and redox metabolism

#### Multivariate profiling of antioxidant strategies and environmental drivers

**Fig. 1:**
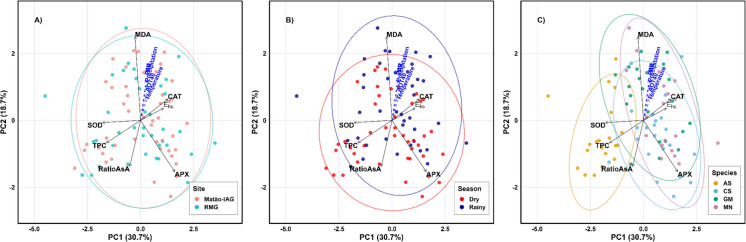
Principal Component Analysis (PCA) of leaf biochemical variables across two study sites, two seasons, and four tree species, with environmental variables fitted as supplementary vectors. Black vectors indicate biochemical variables: malondialdehyde (MDA), superoxide dismutase (SOD), catalase (CAT), ascorbate peroxidase (APX), ascorbate (AsA) redox ratio [AsA/(AsA + dehydroascorbate, DHA)], glutathione (GSH) half-cell reduction potential (E_hc_), and total phenolic content (TPC). Blue dashed vectors represent supplementary environmental predictors: temperature, precipitation, AOT40 (Accumulated ozone exposure over a threshold of 40 ppb), PAR (photosynthetically active radiation), and UV (ultraviolet) irradiance. **A **Samples grouped by site (Morro Grande Forest Reserve [RMG]; Biosciences Institute Forest Reserve [Matão-IAG]), **B** by season (Dry/Rainy), and **C** by species (*Alchornea sidifolia* [AS], *Casearia sylvestris* [CS], *Guarea macrophylla* [GM], *Machaerium nyctitans* [MN]). Ellipses represent 95% confidence intervals for each grouping

PCA was used to assess global variation in redox-related variables across species, sites, and seasons (Fig. 1). The first two components explained 49.4% of the variance (PC1: 30.7%; PC2: 18.7%). PC1 mainly separated samples with higher SOD and TPC from those with higher APX, CAT, and E_hc_, whereas MDA contributed weakly. PC2 was driven positively by MDA and CAT and negatively by APX, RatioAsA, and TPC. Supplementary environmental vectors aligned mainly with PC2, particularly precipitation, PAR, and UV, while AOT40 and temperature showed weaker positive associations. Site groups largely overlapped (Fig. 1A), whereas seasonal separation was clearer (Fig. 1B), with rainy-season samples associated with MDA and CAT and dry-season samples with higher RatioAsA, APX, and TPC. Species separation was weaker and mainly driven by AS (Fig. 1C). PERMANOVA supported these patterns: species explained the largest fraction of multivariate variation, followed by season, whereas site had no meaningful effect (species: *R*^2^ = 0.2915, *p* = 0.001; season: *R*^2^ = 0.0370, *p* = 0.010; site: *R*^2^ = 0.0033, *p* = 0.956).

RDA further supported the PCA patterns. Measured environmental gradients explained little biochemical variation and were not significant overall (5.1%; *F* = 1.06, *p* = 0.384). The site–season model was also weak and non-significant (4.6%; *F* = 1.29, *p* = 0.185). After controlling for site, season explained a modest but significant fraction of variation (3.7%; *F* = 3.10, *p* = 0.010), whereas site explained little after controlling for season (0.31%; *F* = 0.26, *p* = 0.962). The Site × Season interaction was not significant (*F* = 0.50, *p* = 0.808), and variance partitioning confirmed the limited independent contribution of environmental variables.

Exploratory species-specific Pearson correlations were mostly weak to moderate (Figure S1). MDA showed the most consistent positive associations with environmental variables, strongest in CS (*r* = 0.42–0.64) and weaker in AS, GM, and MN (*r* = 0.05–0.47). TPC was mostly weakly associated with environmental variables, except for negative correlations with AOT40 in AS and CS (*r* = − 0.36 and − 0.49, respectively) and with temperature, precipitation, PAR, and UV in MN (*r* = − 0.42 to − 0.36). Antioxidant-related traits showed species-specific patterns: SOD and E_hc_ were negatively correlated with most environmental variables in AS (*r* = − 0.48 to − 0.30), whereas E_hc_ was positively correlated with all environmental variables in GM (*r* = 0.28–0.47). In CS, APX was negatively correlated with environmental variables (*r* = − 0.39 to − 0.24), while RatioAsA was positively associated with AOT40 (*r* = 0.39). MN showed mostly weak and mixed correlations, with only modest positive associations of APX and CAT with precipitation, PAR, and UV (*r* = 0.15–0.23).

#### Modulation of the ascorbate (AsA) pools

**Fig. 2:**
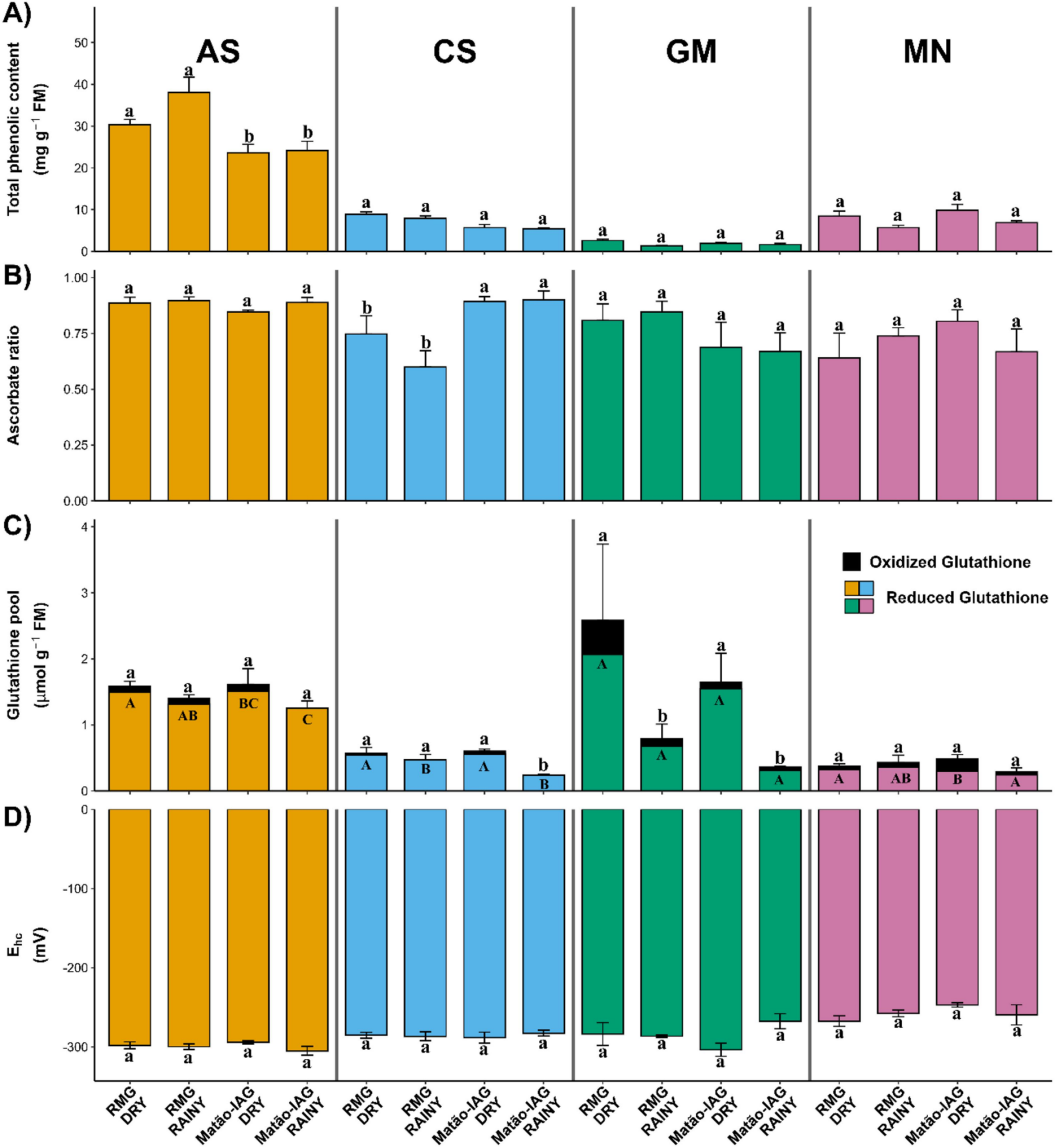
Summary of non-enzymatic antioxidant variables in leaves of four native Atlantic Forest tree species. Panels show **A** total phenolic content, **B** ascorbate redox ratio, **C** glutathione pool size, with reduced forms shown in colored bars and oxidized forms shown in black bars, and **D** glutathione half-cell reduction potential (E_hc_). Columns correspond to *Alchornea sidifolia* (AS), *Casearia sylvestris* (CS), *Guarea macrophylla* (GM), and *Machaerium nyctitans* (MN). Sites are Morro Grande Forest Reserve (RMG) and Biosciences Institute Forest Reserve (Matão-IAG). FM, fresh mass. Data represent mean ± standard error (*n* = 6). Different lowercase letters indicate statistically significant differences among site–season conditions within each species (Sidak-adjusted pairwise comparisons, *p* < 0.05)

AsA responses were species-specific, with site effects expressed mainly as changes in pool size rather than redox partitioning (Fig. 2B; Tables S1–S7). In AS, additive models showed that urban exposure reduced AsA (site *p* = 0.005), while season had only marginal effects on AsA and TotalAsA (*p* = 0.057 and *p* = 0.052, respectively). AsA and TotalAsA were lower at Matão-IAG than at RMG in both the dry season (AsA: 2.56 vs. 4.21 µmol g⁻^1^ FM, − 39%; TotalAsA: 2.99 vs. 4.72, − 37%) and the rainy season (AsA: 3.68 vs. 5.34 µmol g⁻^1^ FM, − 31%; TotalAsA: 4.17 vs. 5.90, − 29%). DHA was invariant (null model; 0.506 µmol g⁻^1^ FM), and RatioAsA showed no supported variation (0.847–0.906; site *p* = 0.167, season *p* = 0.130).

In CS, AsA and TotalAsA showed Site × Season interactions, with maxima at RMG in the dry season (AsA: 1.30 µmol g⁻^1^ FM, *p* = 0.017; TotalAsA: 1.55 µmol g⁻^1^ FM, *p* = 0.031) and lower values in all other site–season combinations. DHA declined from RMG–Dry and RMG–Rainy values of 0.279 and 0.091 µmol g⁻^1^ FM, respectively, to 0.0070 and 0.0023 µmol g⁻^1^ FM at Matão-IAG. Conversely, RatioAsA was higher at Matão-IAG than at RMG in both seasons (site-only model; 0.897 vs. 0.673; site *p* < 0.001).

In GM, AsA showed a Site × Season interaction (*p* = 0.030), with the highest value at RMG in the dry season (0.056 µmol g⁻^1^ FM). TotalAsA followed a season-only model and was lower in the rainy than in the dry season (0.044 vs. 0.071 µmol g⁻^1^ FM; *p* = 0.016). DHA showed a marginal threefold increase at Matão-IAG (0.034 vs. 0.011 µmol g⁻^1^ FM; *p* = 0.053), accompanied by a non-significant tendency toward lower RatioAsA (0.679 vs. 0.827; *p* = 0.073).

In MN, AsA and TotalAsA followed additive models, with strong site effects (site *p* < 0.001) and no supported seasonal effect (season *p* = 0.122–0.141). Both pools were lower at Matão-IAG than at RMG in the dry season (AsA: 0.056 vs. 0.085 µmol g⁻^1^ FM; TotalAsA: 0.068 vs. 0.152 µmol g⁻^1^ FM) and rainy season (AsA: 0.046 vs. 0.074 µmol g⁻^1^ FM; TotalAsA: 0.050 vs. 0.113 µmol g⁻^1^ FM). DHA was also lower at Matão-IAG (0.015 vs. 0.057 µmol g⁻^1^ FM, − 74%; p = 0.006), whereas RatioAsA was invariant (null model; mean = 0.713).

Across species, RatioAsA was affected by Species (likelihood-ratio, LR, χ^2^ = 10.64, df = 3, *p* = 0.0138) and Species × Site (LR χ^2^ = 13.30, df = 3, *p* = 0.0040), but not by Site, Season, or other interactions (*p* ≥ 0.209). This pattern reflected the site-dependent increase in CS, whereas the other species showed no supported site effect. Tukey-adjusted comparisons detected one significant contrast, with AS exceeding CS at RMG in the rainy season (*p* = 0.0196).

#### Glutathione homeostasis and the maintenance of cellular redox potential

While ascorbate responses were largely site-driven, glutathione homeostasis was shaped mainly by season and Site × Season interactions (Fig. 2C, Tables S8–S14). In AS, reduced GSH followed a season-only model, although the seasonal effect was not supported (*p* = 0.145; dry: 1.58 µmol g⁻^1^ FM; rainy: 1.33 µmol g⁻^1^ FM). GSSG varied by season (*p* = 0.003), but not by site (*p* = 0.150), and was approximately twofold higher in the dry season at both sites (RMG: 0.136 vs. 0.066 µmol g⁻^1^ FM; Matão-IAG: 0.096 vs. 0.046 µmol g⁻^1^ FM; dry vs. rainy). TotalGSH showed only marginal seasonal variation (*p* = 0.078; 1.69 vs. 1.39 µmol g⁻^1^ FM; − 18%), and E_hc_ showed no supported seasonal effect (*p* = 0.152).

In CS, GSH, GSSG, and TotalGSH showed Site × Season interactions (*p* = 0.015, *p* = 0.002, and *p* < 0.001, respectively). These reflected larger rainy-season declines at Matão-IAG (GSH: 0.616 vs. 0.212 µmol g⁻^1^ FM, − 66%; TotalGSH: 0.655 vs. 0.216 µmol g⁻^1^ FM, − 67%; GSSG: 0.040 vs. 0.004 µmol g⁻^1^ FM, − 90%) than at RMG (GSH: 0.542 vs. 0.470 µmol g⁻^1^ FM, − 13%; TotalGSH: 0.569 vs. 0.501 µmol g⁻^1^ FM, − 12%; GSSG: 0.027 vs. 0.014 µmol g⁻^1^ FM, − 49%). Despite these pool-size changes, E_hc_ was invariant (null model; mean = − 287 mV).

In GM, seasonality strongly affected glutathione status. GSH and TotalGSH declined in the rainy season (*p* < 0.001), decreasing by 77–78% (GSH: 2.21 vs. 0.48 µmol g⁻^1^ FM; TotalGSH: 2.40 vs. 0.56 µmol g⁻^1^ FM). GSSG also declined seasonally (*p* = 0.023), decreasing by 57% at both sites (RMG: 0.262 vs. 0.112 µmol g⁻^1^ FM; Matão-IAG: 0.140 vs. 0.060 µmol g⁻^1^ FM). E_hc_ showed a marginal seasonal shift toward less negative values in the rainy season (*p* = 0.059; − 295 vs. − 274 mV).

In MN, reduced GSH showed marginal site dependence (*p* = 0.082), with lower values at Matão-IAG than at RMG (0.270 vs. 0.439 µmol g⁻^1^ FM; − 39%). GSSG and TotalGSH showed Site × Season interactions (*p* = 0.001 and *p* = 0.004, respectively). GSSG was higher at Matão-IAG than at RMG in the dry season (0.195 vs. 0.063 µmol g⁻^1^ FM; + 209%) but lower in the rainy season (0.045 vs. 0.103 µmol g⁻^1^ FM; − 56%). TotalGSH showed the same crossover, with higher values at Matão-IAG in the dry season (0.598 vs. 0.378 µmol g⁻^1^ FM; + 58%) and higher values at RMG in the rainy season (0.619 vs. 0.288 µmol g⁻^1^ FM; + 115%). E_hc_ was invariant (null model; mean = − 258 mV).

Across species, E_hc_ differed significantly (F(3,75) = 22.87, *p* = 1.29 × 10^−10^), with context-dependent variation indicated by a Species × Site × Season interaction (F(3,75) = 2.82, *p* = 0.0446). MN consistently showed the least negative E_hc_ values (Fig. 2D).

#### Total phenolic content (TPC)

TPC differed strongly among species (Fig. 2A, Table S15), and the magnitude of these differences varied with site and season, as indicated by a significant Species × Site × Season effect (*p* = 0.025). Despite this interaction, Tukey-adjusted EMMs showed the same ranking across all Site × Season combinations, with AS highest, GM lowest, and CS and MN intermediate and not significantly different. At the within-species level, AS showed a clear site effect (*p* = 0.001), with higher TPC at RMG than at Matão-IAG in both seasons (31.62–38.10 vs. 24.16–26.70 mg g⁻^1^ FM; dry vs. rainy). CS followed the same general pattern, consistent with an additive model with a significant site effect (*p* = 0.010) but no supported seasonal effect (*p* = 0.143), with higher TPC at RMG than at Matão-IAG (7.90–8.88 vs. 5.44–6.91 mg g⁻^1^ FM), although adjusted comparisons among the four site–season means were not significant. In GM, TPC was stable across conditions (null model; mean = 2.01 mg g⁻^1^ FM). In MN, TPC was higher in the dry season than in the rainy season at both sites (RMG: 8.44 vs. 5.70 mg g⁻^1^ FM; Matão-IAG: 9.85 vs. 7.68 mg g⁻^1^ FM), consistent with an additive model in which season was significant (*p* = 0.019) but site was not (*p* = 0.106), although adjusted comparisons did not distinguish individual site–season means.

#### Enzymatic antioxidant activity

**Fig. 3:**
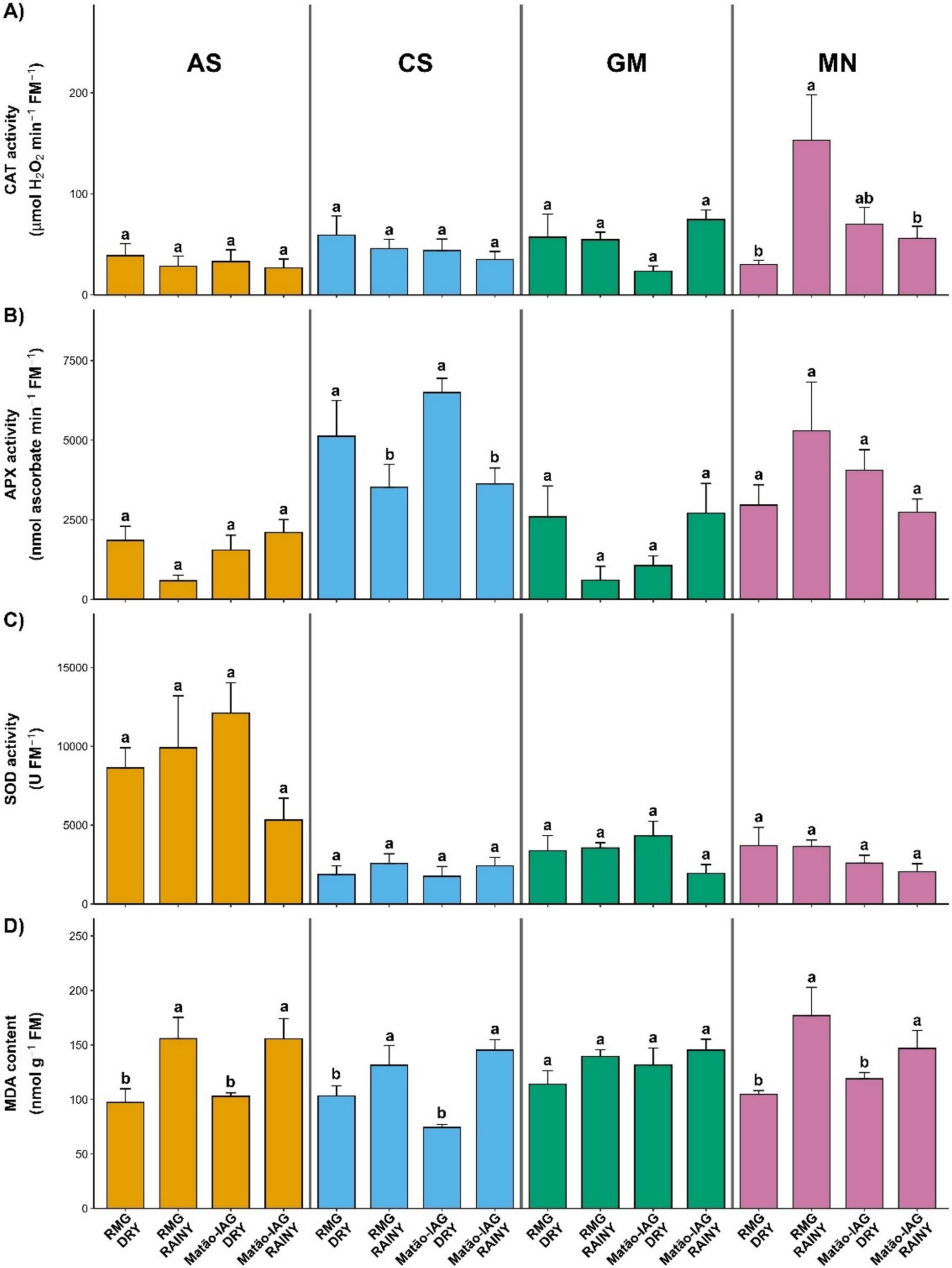
Summary of oxidative damage and enzymatic antioxidant activity in leaves of four native Atlantic Forest tree species. Panels show **A** catalase (CAT) activity, **B** ascorbate peroxidase (APX) activity, **C** superoxide dismutase (SOD) activity, and **D** lipid peroxidation measured as malondialdehyde (MDA) content. Columns correspond to *Alchornea sidifolia* (AS), *Casearia sylvestris* (CS), *Guarea macrophylla* (GM), and *Machaerium nyctitans* (MN). Sites are Morro Grande Forest Reserve (RMG) and Biosciences Institute Forest Reserve (Matão-IAG). Data represent mean ± standard error (*n* = 6). Different lowercase letters indicate statistically significant differences among site–season conditions within each species (Sidak-adjusted pairwise comparisons, *p* < 0.05)

Enzymatic antioxidant activity differed strongly among species (Fig. 3A–C; Tables S16–S18). In AS, SOD and CAT were stable across site and season (null models; mean SOD: 9,170 U g⁻^1^ FM, the highest constitutive activity among species; mean CAT: 31.9 µmol H₂O₂ min⁻^1^ g⁻^1^ FM). APX followed a Site × Season interaction (*p* = 0.026), with similar dry-season predictions between sites (RMG: 1.85; Matão-IAG: 1.55 µmol AsA oxidized min⁻^1^ g⁻^1^ FM) but divergent rainy-season predictions (RMG: 0.583; Matão-IAG: 2.10 µmol AsA oxidized min⁻^1^ g⁻^1^ FM), although post hoc comparisons were not significant.

In CS, SOD and CAT also showed no site or season effects (null models; 2,160 U g⁻^1^ FM and 46.5 µmol H₂O₂ min⁻^1^ g⁻^1^ FM, respectively). APX was higher in the dry than in the rainy season (5.67 vs. 3.58 µmol AsA oxidized min⁻^1^ g⁻^1^ FM; *p* < 0.01).

In GM, APX was invariant (null model; 1.85 µmol AsA oxidized min⁻^1^ g⁻^1^ FM). CAT followed a Site × Season structure, with a significant season effect (*p* = 0.042) and marginal interaction (*p* = 0.068): dry-season predictions were higher at RMG than at Matão-IAG (57.0 vs. 23.5 µmol H₂O₂ min⁻^1^ g⁻^1^ FM), whereas rainy-season predictions were higher at Matão-IAG than at RMG (74.5 vs. 54.5 µmol H₂O₂ min⁻^1^ g⁻^1^ FM). SOD followed a season-only model but without a supported seasonal effect (3950 vs. 2540 U g⁻^1^ FM; *p* = 0.096).

In MN, APX was invariant (null model; 3.81 µmol AsA oxidized min⁻^1^ g⁻^1^ FM). SOD differed by site (3680 vs. 2320 U g⁻^1^ FM at RMG and Matão-IAG, respectively; *p* = 0.044), although post hoc comparisons did not distinguish Site × Season means. CAT showed a Site × Season interaction (*p* < 0.001) and season effect (*p* = 0.011), with higher activity at Matão-IAG in the dry season (70.1 vs. 30.1 µmol H₂O₂ min⁻^1^ g⁻^1^ FM) but higher activity at RMG in the rainy season (153.0 vs. 56.0 µmol H₂O₂ min⁻^1^ g⁻^1^ FM).

#### Lipid peroxidation

 Season was the main factor associated with variation in MDA levels (Fig. 3D, Table S19). AS, CS, and MN showed a consistent pattern of higher MDA values in the rainy season relative to the dry season. The increase was significant in all three species, rising from 112 to 162 nmol g⁻^1^ FM in MN (*p* < 0.001), from 97 to 156 nmol g⁻^1^ FM in AS (*p* < 0.001), and from 90 to 138 nmol g⁻^1^ FM in CS (*p* < 0.001). GM followed the same trend, with MDA increasing from 124 to 142 nmol g⁻^1^ FM, but the difference was not significant (*p* = 0.103).

### Leaf anatomical damage in light microscopy

**Fig. 4:**
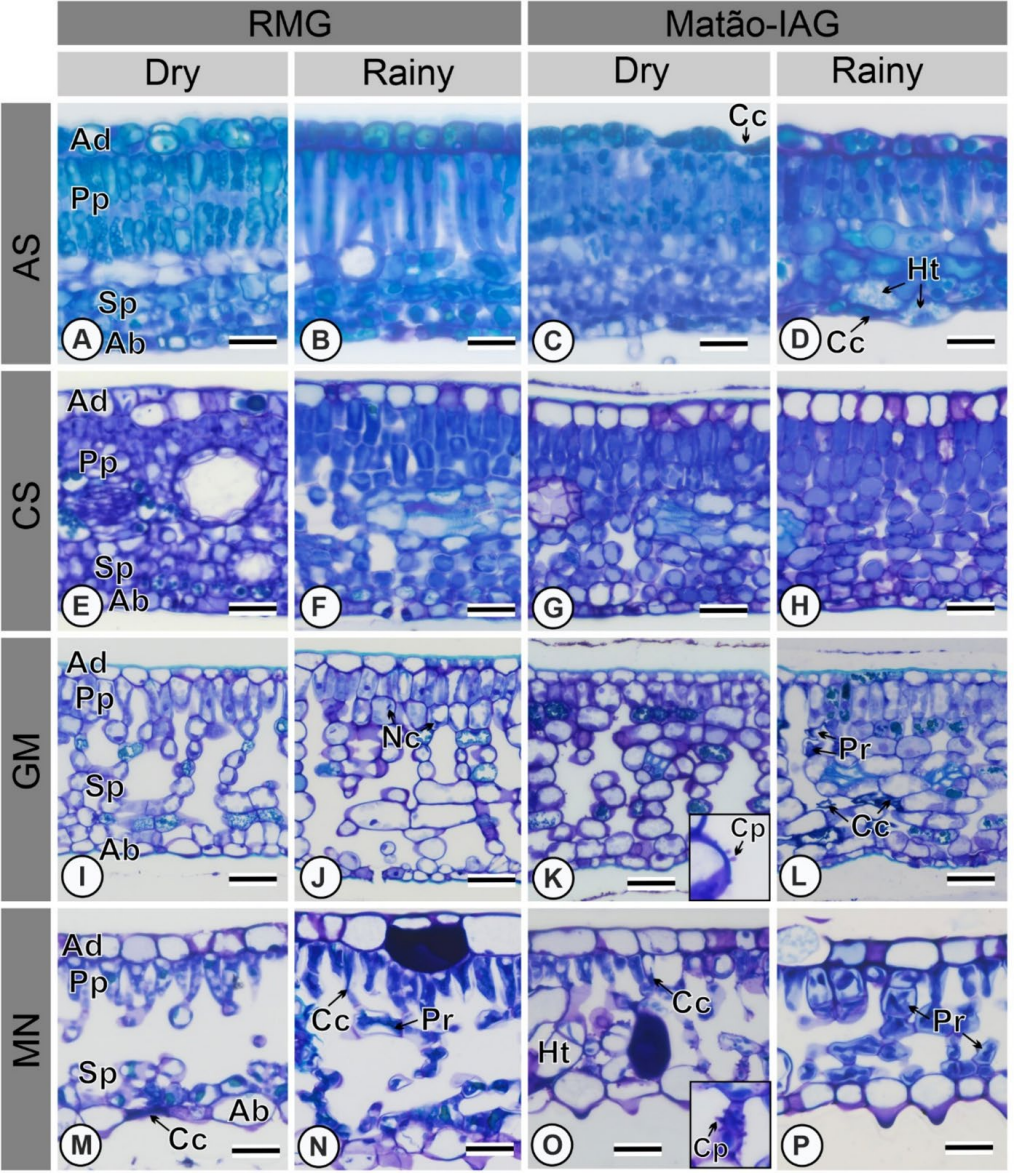
Anatomical damage in leaves of *Alchornea sidifolia* (AS, A–D), *Casearia sylvestris* (CS, E–H), *Guarea macrophylla* (GM, I–L), and *Machaerium nyctitans* (MN, M–P) from Morro Grande Forest Reserve (RMG) and Biosciences Institute Forest Reserve (Matão-IAG) under dry and rainy season conditions. Ab, abaxial epidermis; Ad, adaxial epidermis; Cc, cell collapse; Cp, cell wall protrusions; Ht, cell hypertrophy; Nc, nuclear collapse; Pa, accumulation of phenolic compounds; Pr, protoplast retraction; Sp, spongy parenchyma. Scale bars: A–D = 20 µm; E–H = 30 µm; I–L = 50 µm; M–P = 30 µm

All species had leaves with a single-layered epidermis and dorsiventral mesophyll (Fig. 4A–P). AS (Fig. 4A–D) and CS (Fig. 4E–H) showed compact mesophyll with few intercellular spaces, whereas GM (Fig. 4I–L) and MN (Fig. 4M–P) had more porous mesophyll with extensive intercellular spaces. Mesophyll type and organization were consistent within each species across sites and seasons.

Overall, anatomical damage was more pronounced at Matão-IAG than at RMG (Fig. 4). At RMG, damage occurred only in GM and MN and was characterized by cell collapse and protoplast retraction (Fig. 4I, J, M, and N). At Matão-IAG, all species except CS (Fig. 4G, H) showed damage in both seasons. In AS, epidermal cell collapse occurred in both seasons, and mesophyll cell hypertrophy in the rainy season (Fig. 4C and D). In GM, cell wall protrusions occurred in the dry season, whereas cell collapse with protoplast retraction occurred in the rainy season (Fig. 4K and L). MN showed the greatest tissue-level sensitivity among the studied species, with the most extensive damage, including cell wall protrusions and cell collapse in the dry season and widespread protoplast retraction in the rainy season (Fig. 4M–P). In the porous-mesophyll species, GM and MN, damage was more pronounced in the rainy season at both sites (Fig. 4J, L, N, and P).

### Cell death

**Fig. 5:**
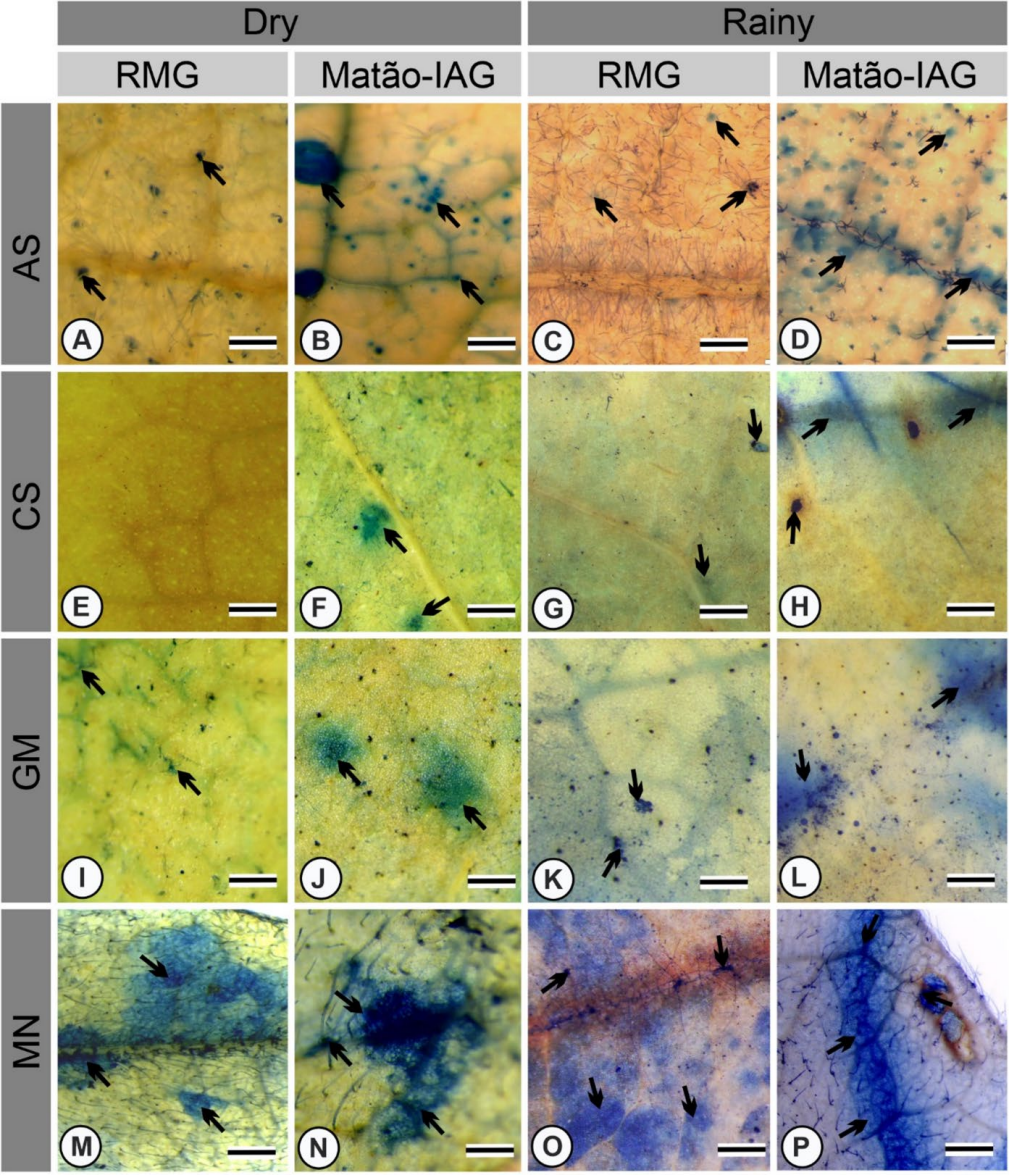
Evans Blue staining indicating cell death in leaves of *Alchornea sidifolia* (AS, A–D), *Casearia sylvestris* (CS, E–H), *Guarea macrophylla* (GM, I–L), and *Machaerium nyctitans* (MN, M–P) collected at Morro Grande Forest Reserve (RMG) and Biosciences Institute Forest Reserve (Matão-IAG) during the dry and rainy seasons. Blue staining indicates loss of membrane integrity and a positive cell death reaction (arrows). Scale bars: A–P = 0.4 cm

The cell death assay was positive in all four species at both sites and in both seasons (Fig. 5A–P), but reaction intensity and spatial extent varied among species, sites, and seasons. MN showed the strongest response, with positivity across larger areas of the leaf blade (Fig. 5M–P), whereas AS, CS, and GM showed weaker reactions (Fig. 5A–L).

Site-specific patterns were also evident. At RMG, reactions were generally confined to small, isolated regions, except in MN, which showed extensive positivity (Fig. 5M and N). At Matão-IAG, all species showed more intense and widespread staining across the leaf blade (Fig. 5C, D, G, H, K, L, O, and P). At this site, AS and MN also showed greater spatial positivity in the rainy season (Fig. 5C, D, O, and P).

## Discussion

This study examined how four native Atlantic Forest tree species regulate oxidative stress across contrasting seasons and environmental conditions. By integrating redox biochemistry with anatomical indicators of damage and cell death, we linked antioxidant regulation to tissue-level outcomes under field conditions. Three main patterns emerged. First, multivariate variation in redox-related variables was driven mainly by species identity and season, with little site separation. Second, contrary to our expectation, lipid peroxidation was more consistently associated with rainy-season than dry-season conditions. Third, despite weak biochemical site effects, microscopy revealed more frequent and spatially extensive damage and cell death at the urban site, with greater expression in the rainy season. Because antioxidant regulation differed strongly among species, whereas oxidative and anatomical damage were more consistently structured by season and site, we first discuss species-specific redox strategies, followed by seasonality and urban exposure as shared drivers of oxidative stress.

### Species-specific strategies

#### Alchornea sidifolia (AS)

AS showed a comparatively buffered redox phenotype associated with constitutive antioxidant capacity and compact mesophyll architecture, rather than with strong enzymatic plasticity. This phenotype is characterized by a comparatively large AsA pool, a stable, reducing GSH redox potential, consistently high SOD activity, and compact mesophyll.

Large AsA pools support H₂O₂ detoxification within the AsA–GSH hub (Smirnoff & Wheeler, 2024). At the urban site, where O₃ metrics were highest, AS showed lower AsA and TotalAsA while maintaining a high RatioAsA (> 0.8), indicating pool depletion without loss of redox partitioning (Anselmo-Moreira et al., 2026). This pattern suggests increased antioxidant demand with sufficient regeneration to preserve AsA redox status (Foyer & Kunert, 2024) as reported under ozone stress in *Vicia faba* and tobacco (Dai et al., 2019; Turc et al., 2021). In parallel, AS maintained a highly reducing glutathione state (E_hc_ − 292 to − 300 mV) across conditions, consistent with maintenance of buffering conditions compatible with AsA recycling even when total pools declined (Müller-Schüssele et al., 2021; Terai et al., 2020).

Enzymatically, AS showed consistently high constitutive SOD activity compared to the other species, while both SOD and CAT remained stable across conditions and APX showed limited condition-dependent variation. This pattern suggests greater reliance on constitutive antioxidant capacity than on strong enzyme induction. Non-enzymatic antioxidants likely complement this background. Consistently high TPC supports a contribution of phenolics to basal buffering capacity, and lower levels at Matão-IAG are compatible with partial consumption under higher oxidative demand. Such declines have been reported under O_3_ exposure, likely reflecting direct oxidation or use during ROS scavenging (Marchica et al., 2020; Rao & Zheng, 2025). This biochemical background is consistent with a possible contribution of leaf structure, as compact mesophyll may help restrict internal diffusion of O_3_ and other pollutants, thereby limiting ROS formation within the tissue (Xu et al., 2023).

Despite this buffered phenotype, AS showed clear anatomical damage at Matão-IAG, including epidermal collapse in both seasons and mesophyll hypertrophy in the rainy season. Together with greater cell death and higher rainy-season lipid peroxidation, these responses indicate that urban oxidant exposure combined with rainy-season conditions can exceed constitutive buffering capacity. The predominance of epidermal damage suggests that injury may develop near the stomatal/apoplastic interface, where pollutant entry and early ROS formation generate localized oxidative hotspots (Hasan et al., 2021; Morales et al., 2021). Thus, AS showed a comparatively buffered redox phenotype, but only partial protection against tissue-level damage at Matão-IAG.

#### Casearia sylvestris (CS)

CS was the most structurally resilient species, uniquely maintaining leaf integrity at the urban site in both seasons. This tolerance appears to involve an antioxidant strategy that preserves a reduced redox state despite shifts in pool size and may also be influenced by compact mesophyll architecture.

AsA pools varied with site and season, and RatioAsA was higher at Matão-IAG in both seasons because DHA remained very low, indicating maintenance of a strongly reduced AsA state despite changes in pool size. Glutathione showed a similar pattern: despite a marked rainy-season contraction at Matão-IAG, E_hc_ remained reduced and invariant (≈ − 287 mV), indicating preservation of GSH redox status. Because APX consumes AsA during H₂O₂ detoxification and generates DHA that is typically recycled through GSH-dependent steps, the comparatively high APX capacity of CS, together with high RatioAsA and stable E_hc_, is consistent with close functional coupling of the AsA–GSH system, supporting peroxide detoxification without a persistent shift toward oxidation (Corpas et al., 2024). Similar to AS, phenolics appear to play a complementary role. Higher TPC at RMG than at Matão-IAG, together with predominantly negative associations with AOT40 and PAR/UV, suggests that phenolic levels declined rather than increased under higher urban oxidative load. This pattern is compatible with partial phenolic consumption or oxidation during stress, implying that these compounds support, rather than define, resilience of CS to tissue-level injury under urban exposure.

Despite this buffered redox phenotype, CS still showed rainy-season increases in MDA and positive cell death staining at Matão-IAG, indicating stress activation. However, unlike the other species, it showed no anatomical damage in either season, indicating that stress did not progress to tissue-level collapse. Although both CS and AS have compact mesophyll, the epidermal and mesophyll damage in AS indicates that mesophyll compactness alone does not fully explain the resilience of CS. Additional protective mechanisms likely help limit damage propagation under urban exposure. Biogenic volatile organic compound (BVOC) protection is one plausible component, as CS has been reported to emit high levels of BVOCs, particularly sesquiterpenes (Borbon et al., 2026; Costa et al., 2025, 2026; de Araújo et al., 2025). These compounds may attenuate oxidative injury by scavenging O₃ and related oxidants both near the leaf surface and within intercellular spaces after uptake, thereby reducing oxidant penetration and limiting damage to internal tissues (Palmer-Young et al., 2015; Yu et al., 2015).

#### Guarea macrophylla (GM)

GM displayed a seasonally labile antioxidant phenotype, in which non-enzymatic buffering varied more than enzymatic activity, and this lability coincided with pronounced tissue-level vulnerability under urban exposure. The dominant signal was instability in the AsA–GSH redox system: GM showed a pronounced rainy-season contraction of glutathione, with GSH and TotalGSH declining by > 75%. Together with the ~ threefold increase in DHA at Matão-IAG, this pattern suggests that rainy-season demand approached or exceeded the biosynthetic and/or regenerative capacity of the AsA–GSH cycle. Consistent with this, E_hc_ shifted toward less reducing values in the rainy season (− 295 to − 274 mV), indicating high oxidative demand and/or constrained replenishment capacity under O_3_ and high irradiance (Grulke & Heath, 2020). Enzymatic responses were only partly compensatory: CAT increased at the urban site in the rainy season, whereas SOD and APX showed no comparably robust adjustment, suggesting limited capacity to scale detoxification when chemical buffers contracted (Dai et al., 2019). This limitation extended to the phenolic pool. GM had the lowest TPC overall and showed no significant variation across site or season, indicating that bulk phenolics provided neither a strong constitutive buffer nor an inducible layer of protection when AsA–GSH homeostasis became destabilized.

This biochemical limitation was likely amplified by leaf structure. GM has a porous mesophyll with extensive intercellular air spaces, which may increase internal gas diffusion and facilitate deeper oxidant penetration through the apoplast. Under this architecture, depletion of the AsA–GSH buffering system would reduce ROS-quenching capacity, increasing the likelihood that localized oxidative hotspots propagate into broader cellular dysfunction (Yu & Blande, 2022). Consistent with this, GM exhibited rainy-season cell collapse with protoplast retraction, more evident at Matão-IAG, together with more intense and widespread cell death staining at the urban site and a tendency toward higher rainy-season MDA. Together, a more diffusion-permissive mesophyll, seasonally depleted AsA–GSH buffering, and limited enzymatic compensation provide a mechanistic basis for the high vulnerability of GM to oxidative pressure, particularly at the urban site during the rainy season.

#### Machaerium nyctitans (MN)

MN showed the greatest oxidative and tissue-level sensitivity under the studied urban and seasonal exposure conditions, characterized by high oxidative damage, a comparatively oxidized glutathione background, and extensive tissue-level failure. In MN, the ascorbate system maintained redox partitioning despite urban-associated depletion, as RatioAsA remained invariant across site and season even though AsA and TotalAsA were lower at Matão-IAG. However, MN differed markedly in buffering capacity because its absolute AsA pools were small and substantially lower than those of AS and CS. The dominant limitation in MN appears to lie in glutathione homeostasis: it exhibited a consistently less reducing glutathione background than the other species (E_hc_ ≈ − 258 mV), indicating weaker basal redox buffering under sustained oxidative challenge.

MN did not show coordinated enzymatic adjustment consistent with effective compensation. SOD was lower at Matão-IAG, and CAT showed a pronounced seasonal switch, being higher at Matão-IAG in the dry season but strongly induced at RMG in the rainy season. APX did not vary across conditions and, together with depleted AsA pools at Matão-IAG, likely limited AsA-dependent peroxide detoxification when oxidative demand rose. This limitation also extended to the phenolic pool. TPC was intermediate overall but consistently higher in the dry season at both sites, indicating that total phenolics were not maintained under the rainy-season conditions in which oxidative damage became most severe. Thus, although phenolics may have contributed to basal protection, their seasonal decline suggests that this layer was insufficient to offset the weak glutathione background and limited enzymatic adjustment during periods of higher oxidative burden.

Consistent with this biochemical profile, MN showed a strong rainy-season increase in MDA and the most extensive anatomical damage, together with intense and widespread cell death staining, especially at the urban site. Its porous mesophyll likely amplified these outcomes by facilitating internal oxidant diffusion and the spread of apoplastic oxidative hotspots, increasing the likelihood that localized damage escalated into broad tissue impairment when chemical pools were small and enzymatic compensation was incomplete.

### Seasonal structure of oxidative responses

Season was the most consistent driver of oxidative responses across species. Lipid peroxidation increased in the rainy season in AS, CS, and MN, with the same trend in GM, and this coincided with more extensive anatomical damage and broader cell death staining. By contrast, antioxidant regulation did not converge on a common seasonal pattern: enzymatic activities were largely species- and context-dependent, and univariate models did not support consistent seasonal induction of SOD, CAT, or APX across taxa. Although the multivariate structure placed dry-season samples closer to higher APX and a more reduced AsA redox ratio, the strongest damage signal was associated with rainy-season conditions.

This pattern contrasts with our expectation that oxidative burden would peak in the dry season, as commonly predicted when seasonal water limitation is the dominant stressor. In the MASP Atlantic Forest, however, the winter dry period may involve reduced rainfall without sustained physiological drought (Metzger et al., 2006; Morellato et al., 2000). Under these conditions, the rainy season may instead concentrate oxidative forcing because it combines higher PAR/UV and warmer temperatures, increasing photooxidative pressure and the likelihood of membrane peroxidation when antioxidant and photoprotective capacity are exceeded (Lu et al., 2017).

This context dependence is consistent with earlier Atlantic Forest studies but also indicates that the seasonal maximum of oxidative damage is not fixed across regions or species sets. Aguiar-Silva et al. (2016), working with three abundant semideciduous Atlantic Forest species (*Astronium graveolens*, *Croton floribundus* and *Piptadenia gonoacantha*) in the Campinas region, likewise interpreted rainy-season conditions as potentially photooxidative because high light, water availability, and temperature favor photosynthesis and growth. However, they identified the dry season as the period of strongest oxidative imbalance. This contrast may reflect both climatic differences between regions and species-specific responses. Campinas remnants, being more inland, are likely subject to stronger dry-season vapor pressure deficit, whereas our sites, especially RMG, lie in an Atlantic Plateau transition zone influenced by wetter coastal conditions, which may buffer winter water limitation relative to more interior semideciduous fragments (Metzger et al., 2006). In addition, species-specific functional traits, including successional strategy, constitutive antioxidant capacity, and leaf structural control over oxidant diffusion, likely modulate the magnitude and timing of oxidative stress response, as well as other physiological responses (Esposito et al., 2018).

Interestingly, although rainy-season conditions produced the clearest biochemical and tissue-level damage, the dry season left a distinct anatomical signature in the more sensitive taxa. In GM and MN, cell wall protrusions (Cp) were the most consistent dry-season feature, consistent with stress-induced wall remodeling at the apoplastic interface, including localized deposition and oxidative crosslinking of wall components (Günthardt-Goerg et al., 1997). Because fully expanded leaves were sampled from a standardized developmental position, these protrusions may represent a cumulative signature of exposure during leaf expansion and maturation rather than a strictly acute dry-season response (Turc et al., 2021). Similar cell wall projections have been observed in other exposed plants in urban environments and are a common symptom of O_3_ exposure (Fernandes & Moura, 2021; Moura et al., 2018).

### Site effects on redox status and tissue damage

Although multivariate biochemistry showed little separation by site, Matão-IAG displayed a consistent injury phenotype, with more intense and widespread cell death staining and anatomical damage in all species except CS.

The clearest biochemical site effect involved the ascorbate pool. AS and MN showed consistent depletion of AsA and TotalAsA at Matão-IAG across seasons, whereas CS and GM reached their highest pools only at RMG in the dry season, indicating that urban exposure constrained AsA accumulation more consistently than redox partitioning, although CS also showed a site-related shift in RatioAsA. A more selective pattern emerged for phenolics: in AS and CS, lower TPC at Matão-IAG was consistent with reduced non-enzymatic buffering. Together, these patterns are consistent with higher chronic oxidant demand at the urban site, coinciding with more frequent epidermal collapse, protoplast retraction, and widespread cell death. Damage extent was likely modulated by leaf architecture: the porous mesophyll of GM and MN may allow deeper oxidant diffusion and broader damage propagation, whereas the compact mesophyll of AS and CS may restrict damage to more localized regions.

Comparable Atlantic Forest field studies under mixed pollution and seasonal forcing likewise show that oxidative stress and antioxidant strategies are species-dependent, vary seasonally, and are associated with pollution exposure (Aguiar-Silva et al., 2016; Domingos et al., 2015). At the tissue level, they also report pollution-linked damage, including dark stippling in *Astronium graveolens* associated with reduced antioxidant capacity (Domingos et al., 2015) and higher superoxide and H₂O₂ accumulation in the palisade parenchyma and epidermis of more sensitive non-pioneer taxa (Esposito et al., 2018). Unlike our MASP comparison, however, these studies more often reported clearer shifts in biochemical antioxidant traits across seasons and/or among remnants, whereas in our case site effects were clearest in microscopy and cell death and narrower in the biochemical profile (Aguiar-Silva et al., 2016; Brandão et al., 2017).

Together, these responses refine the biomonitoring interpretation of the four species. Rather than serving as direct proxies for pollutant concentrations, AS, CS, GM, and MN may be more appropriately interpreted as complementary effect-based indicators for assessing whether urban oxidant exposure and seasonal photooxidative demand are accommodated by biochemical adjustment or are accompanied by tissue-level injury. This interpretation is consistent with previous Atlantic Forest biomonitoring studies showing that plant responses to air pollution are species-specific and are better resolved through combined biochemical, morphological, and anatomical markers than through single endpoints (Aguiar-Silva et al., 2016; Domingos et al., 2015; Esposito et al., 2018). Thus, the biomonitoring value of these species lies in their paired response profiles: AS/CS define comparatively buffered profiles, with CS showing injury containment and AS showing partial biochemical buffering with localized injury, whereas GM/MN identify more sensitive profiles in which oxidative pressure was more closely associated with structural damage and cell death. This distinction is relevant for biomonitoring because it separates biochemical adjustment from injury progression, helping identify whether urban oxidant and seasonal photooxidative pressure remain within species-specific buffering capacity or are associated with tissue-level injury. From an applied perspective, this type of effect-based information may complement conventional air-quality monitoring by indicating whether pollutant exposure and seasonal environmental conditions are associated with biological stress in native vegetation. This interpretation is aligned with Laurentino et al. (2024), who emphasized that plant biomonitoring in São Paulo can provide useful information on environmental impacts and support public policies aimed at improving environmental quality. After further validation, such species-specific response profiles could support monitoring agencies and environmental managers in identifying vulnerable fragments, prioritizing long-term monitoring sites, and informing conservation and urban-forest management strategies. These potential applications should be considered cautiously, however, because longer time series and controlled exposure experiments, together with O₃ flux or uptake measurements, are still needed before these profiles can be applied operationally.

## Limitations

This study compared two Atlantic Forest remnants during one dry and one rainy season; therefore, the results reflect a specific spatial and temporal window and may not capture broader environmental or interannual variation. Because the design was observational, O₃ exposure, irradiance, temperature, and precipitation covaried across sites and seasons, limiting the isolation of individual stressor effects. In addition, AOT40 and SUM00 represent atmospheric O₃ exposure rather than actual leaf O₃ uptake, which depends on stomatal conductance, leaf microclimate, and phenological state (Fernandes & Moura, 2021; Grulke & Heath, 2020).

The biochemical dataset captured selected redox and oxidative-damage markers but not the full antioxidant network, and TPC did not resolve individual phenolic compounds. Similarly, anatomical observations provided relevant tissue-level evidence of injury, but they were not designed to quantify structural traits such as cuticle thickness, epidermal dimensions, palisade and spongy parenchyma thickness, intercellular-space proportion, or vascular-tissue attributes. Future studies would benefit from quantitative anatomical image analysis combined with leaf surface characterization, including cuticular wax quantity and composition, to better assess how seasonal abiotic variation and urban oxidant exposure affect both internal leaf tissues and protective surface traits. Integration with gas exchange, chlorophyll fluorescence, water status, and stomatal conductance would further improve the interpretation of tissue-level damage and pollutant uptake. Finally, because only four abundant native species were evaluated, broader generalization across Atlantic Forest tree communities requires additional taxa and multi-year sampling.

## Conclusion

This study integrated redox-related variables with anatomical damage and cell death indicators to assess oxidative-stress regulation in four native Atlantic Forest tree species across contrasting sites and seasons. The results distinguish relative sensitivity and resilience to oxidative stress associated with urban atmospheric pollution and seasonal abiotic variability among the studied species and provide evidence relevant to conservation and biomonitoring under increasing urban pressure. Specifically, AS, CS, GM, and MN may contribute complementary effect-based profiles for assessing whether urban O₃-related oxidant pressure and seasonal photooxidative demand are associated mainly with biochemical adjustment or are accompanied by tissue-level injury and cell death.

- Hypothesis (i) was supported: AS and CS showed greater redox stability than GM and MN, although through different strategies. AS combined high constitutive SOD activity, large AsA pools, and high TPC, consistent with strong but incomplete protection. CS showed the most effective stress containment, maintaining a reduced redox state, high APX capacity, and minimal anatomical damage. In contrast, GM and MN combined porous mesophyll with weaker chemical buffering, with GM showing rainy-season limitation of the AsA–GSH hub and MN showing the least reducing E_hc_, limited enzymatic plasticity, and the strongest anatomical damage and cell death phenotype.
- Hypothesis (ii) was not supported, as oxidative damage was greater in the rainy than in the dry season. This indicates that, at the studied sites, the warm, high-irradiance period imposed the dominant oxidative load, likely through combined photooxidative pressure and elevated oxidant exposure.
- Hypothesis (iii) was partially supported: the urban site imposed a higher effective oxidative challenge, expressed mainly as antioxidant-pool depletion and anatomical damage rather than coordinated enzyme induction or a uniform redox shift.

Future studies should expand characterization of chemical and structural defenses using metabolomics to profile phenolics, including flavonoids and related phenylpropanoids, together with complementary measurements of BVOC emission profiles, cuticular wax quantity and composition, and quantitative anatomical traits. In a biome increasingly exposed to urban and climatic pressures, these approaches will help clarify the chemical and structural basis of species-specific resilience by characterizing defense components not captured by the bulk redox metrics and qualitative anatomical assessments used here. An important next step will be to determine whether these antioxidant and structural strategies represent stable species-level traits, plastic responses to local exposure, or acclimation to chronic urban stress. Integrating metabolomic, volatile-emission, and redox datasets with whole-plant performance traits would also strengthen the ecological interpretation of stress biomarkers and, after further validation, support their use by monitoring agencies and environmental managers to inform biomonitoring, conservation planning, restoration, and urban forest management.

## Supplementary Information

Below is the link to the electronic supplementary material.

ESM 1 (DOCX 2.15 MB)

## Data Availability

The data supporting the findings of this study are available within the article and its Supplementary Information. Additional data generated and/or analyzed during the current study are available from the corresponding author on reasonable request.
