# Supplementary material for: Antioxidant strategies of four native tree species across seasons in urban and peri-urban Atlantic Forest remnants
Source: Environ Monit Assess. 2026 Jul 23;198(8):869. doi: 10.1007/s10661-026-15732-0 (PMC13395913; doi:10.1007/s10661-026-15732-0)
Supplement: Supplementary file 1 — (DOCX 2.15 MB) [file 10661_2026_15732_MOESM1_ESM.docx]

**Supplementary Material**

**Antioxidant strategies of four native tree species across seasons in urban and peri-urban Atlantic Forest remnants**


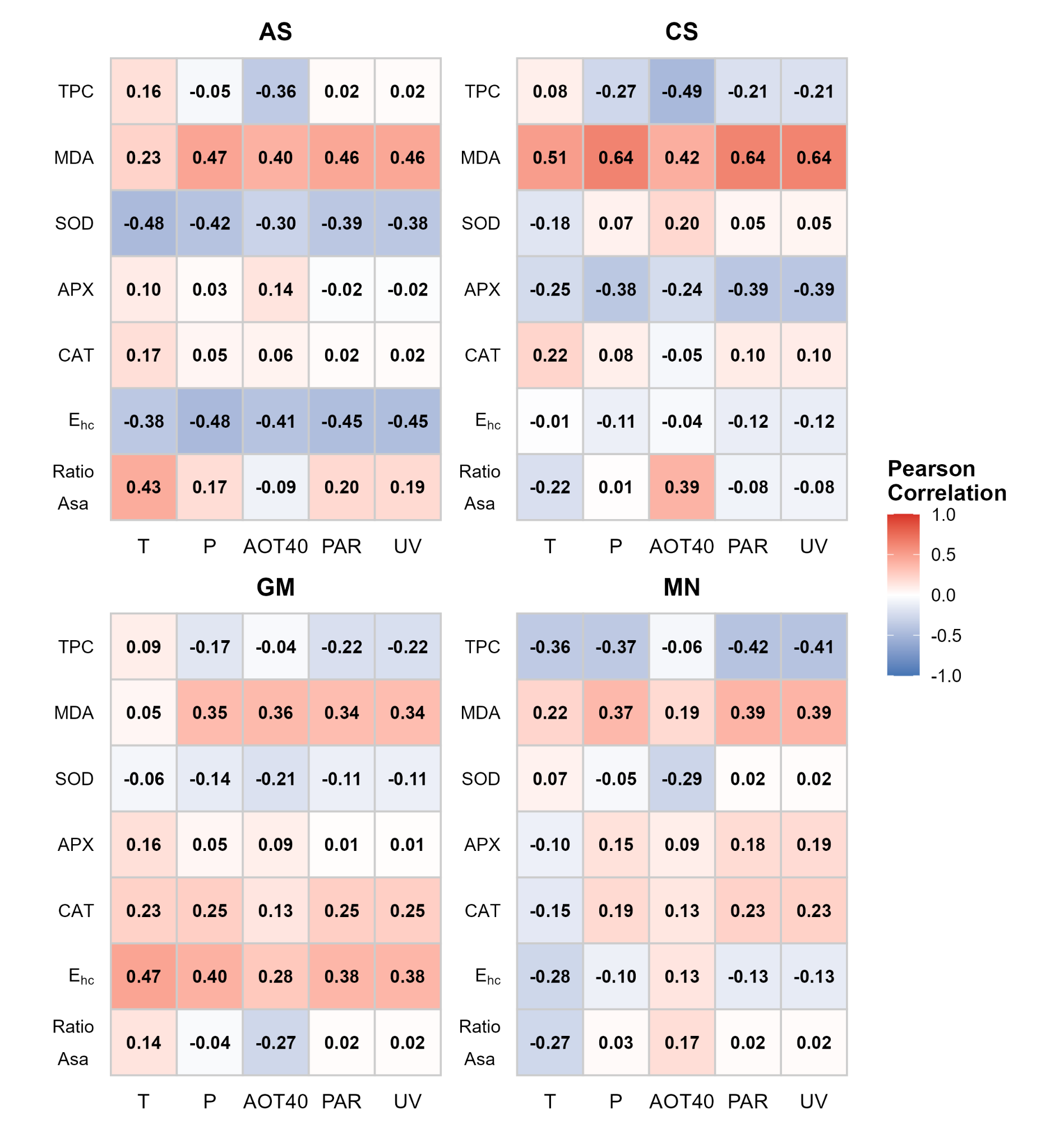


Figure S1. Heatmaps of Pearson correlation coefficients (r) relating leaf biochemical markers to environmental drivers for four native Atlantic Forest tree species. The color scale indicates the strength and direction of the linear relationship: red represents positive correlations (values approaching 1), while blue indicates negative correlations (values approaching -1). AS, *Alchornea sidifolia*; CS, *Casearia sylvestris*; GM, *Guarea macrophylla*; MN, *Machaerium nyctitans*. MDA, malondialdehyde; TPC: total phenolic content; SOD, superoxide dismutase; APX, ascorbate peroxidase; CAT, catalase; Glutathione half-cell reduction potential (E_hc_); RatioAsA, ascorbate redox state. T, temperature; P, precipitation. AOT40, accumulated ozone exposure over a threshold of 40 ppb; PAR, photosynthetically active radiation; UV, ultraviolet radiation index.

Table S1. Influence of site and season on reduced ascorbate (AsA) levels in *Alchornea sidifolia* (AS), *Casearia sylvestris* (CS), *Guarea macrophylla* (GM), and *Machaerium nyctitans* (MN). Values indicate the Akaike Information Criterion (AIC) and significance values (p-values) derived from Linear (LM) and Generalized Linear Models (GLM).

| Species | Model (family; link) | Formula (structure) | AIC | p-values |
| --- | --- | --- | --- | --- |
| AS | LM (Gaussian; identity) | AsA ~ Site + Season | 90.704 | Site = 0.005; Season = 0.057 |
| CS | GLM (Gamma; log) | AsA ~ Site * Season | −31.739 | Site < 0.001; Season < 0.001; Site×Season = 0.017 |
| GM | LM (Gaussian; identity) | AsA ~ Site * Season | −125.33 | Site = 0.001; Season < 0.001; Site×Season = 0.030 |
| MN | LM (Gaussian; identity) | AsA ~ Site + Season | −113.98 | Site < 0.001; Season = 0.141 |

Table S2. Model-predicted reduced ascorbate (AsA) levels in leaves of *Alchornea sidifolia* (AS), *Casearia sylvestris* (CS), *Guarea macrophylla* (GM), and *Machaerium nyctitans* (MN) across site × season combinations. Values are fitted means derived from the best-supported model selected separately for each species (Table S1). RMG, Morro Grande Forest Reserve; Matão-IAG, Biosciences Institute Forest Reserve. Values are expressed as µmol g⁻¹ fresh mass (FM).

| Species | RMG Dry | Matão-IAG Dry | RMG Rainy | Matão-IAG Rainy |  |
| --- | --- | --- | --- | --- | --- |
| AS | 4.21 | 2.56 | 5.34 | 3.68 | |
| CS | 1.3 | 0.068 | 0.165 | 0.044 | |
| GM | 0.056 | 0.038 | 0.034 | 0.03 | |
| MN | 0.085 | 0.056 | 0.074 | 0.046 | |

Table S3. Influence of site and season on dehydroascorbate (DHA) levels in *Alchornea sidifolia* (AS), *Casearia sylvestris* (CS), *Guarea macrophylla* (GM), and *Machaerium nyctitans* (MN). Values indicate the Akaike Information Criterion (AIC) and significance values (p-values) derived from Linear (LM) and Generalized Linear Models (GLM).

| Species | Model (family; link) | Formula (structure) | AIC | p-values |
| --- | --- | --- | --- | --- |
| AS | LM (Gaussian; identity) | DHA ~ 1 (null) | 1.059 | — |
| CS | GLM (Gamma; log) | DHA ~ Site + Season | −111.23 | Site < 0.001; Season < 0.001 |
| GM | GLM (Gamma; log) | DHA ~ Site | −115.03 | Site = 0.053 |
| MN | GLM (Gamma; log) | DHA ~ Site | −105.55 | Site = 0.006 |

Table S4. Model-predicted dehydroascorbate (DHA) levels in leaves of *Alchornea sidifolia* (AS), *Casearia sylvestris* (CS), *Guarea macrophylla* (GM), and *Machaerium nyctitans* (MN) across site × season combinations. Values are fitted means derived from the best-supported model selected separately for each species (Table S3). RMG, Morro Grande Forest Reserve; Matão-IAG, Biosciences Institute Forest Reserve. Values are expressed as µmol g⁻¹ fresh mass (FM).

| Species | RMG Dry | Matão-IAG Dry | RMG Rainy | Matão-IAG Rainy |  |
| --- | --- | --- | --- | --- | --- |
| AS | 0.506 | 0.506 | 0.506 | 0.506 | |
| CS | 0.279 | 0.007 | 0.091 | 0.0023 | |
| GM | 0.011 | 0.034 | 0.011 | 0.034 | |
| MN | 0.057 | 0.015 | 0.057 | 0.015 | |

Table S5. Influence of site and season on total ascorbate (TotalAsA) levels in *Alchornea sidifolia* (AS), *Casearia sylvestris* (CS), *Guarea macrophylla* (GM), and *Machaerium nyctitans* (MN). Values indicate the Akaike Information Criterion (AIC) and significance values (p-values) derived from Linear (LM) and Generalized Linear Models (GLM).

| Species | Model (family; link) | Formula (structure) | AIC | p-values |
| --- | --- | --- | --- | --- |
| AS | LM (Gaussian; identity) | TotalAsA ~ Site + Season | 91.928 | Site = 0.005; Season = 0.052 |
| CS | GLM (Gamma; log) | TotalAsA ~ Site * Season | −23.487 | Site < 0.001; Season < 0.001; Site×Season = 0.031 |
| GM | LM (Gaussian; identity) | TotalAsA ~ Season | −87.437 | Season = 0.016 |
| MN | GLM (Gamma; log) | TotalAsA ~ Site + Season | −77.279 | Site < 0.001; Season = 0.122 |

Table S6. Model-predicted total ascorbate (TotalAsA) levels in leaves of *Alchornea sidifolia* (AS), *Casearia sylvestris* (CS), *Guarea macrophylla* (GM), and *Machaerium nyctitans* (MN) across site × season combinations. Values are fitted means derived from the best-supported model selected separately for each species (Table S5). RMG, Morro Grande Forest Reserve; Matão-IAG, Biosciences Institute Forest Reserve. Values are expressed as µmol g⁻¹ fresh mass (FM).

| Species | RMG Dry | Matão-IAG Dry | RMG Rainy | Matão-IAG Rainy |  |
| --- | --- | --- | --- | --- | --- |
| AS | 4.72 | 2.99 | 5.9 | 4.17 | |
| CS | 1.55 | 0.075 | 0.265 | 0.047 | |
| GM | 0.071 | 0.071 | 0.044 | 0.044 | |
| MN | 0.152 | 0.068 | 0.113 | 0.05 | |

Table S7. Influence of site and season on the reduced ascorbate ratio (RatioAsA) in *Alchornea sidifolia* (AS), *Casearia sylvestris* (CS), *Guarea macrophylla* (GM), and *Machaerium nyctitans* (MN). Values indicate the Akaike Information Criterion (AIC) and significance values (p-values) derived from Linear (LM) and Generalized Linear Models (GLM).

| Species | Model (family; link) | Formula (structure) | AIC | p-values |
| --- | --- | --- | --- | --- |
| AS | LM (Gaussian; identity) | RatioAsA ~ Site + Season | −70.989 | Site = 0.167; Season = 0.130 |
| CS | LM (Gaussian; identity) | RatioAsA ~ Site | −19.093 | Site < 0.001 |
| GM | LM (Gaussian; identity) | RatioAsA ~ Site | −5.917 | Site = 0.073 |
| MN | LM (Gaussian; identity) | RatioAsA ~ 1 (null) | −6.666 | — |

Table S8. Influence of site and season on reduced glutathione (GSH) levels in *Alchornea sidifolia* (AS), *Casearia sylvestris* (CS), *Guarea macrophylla* (GM), and *Machaerium nyctitans* (MN). Values indicate the Akaike Information Criterion (AIC) and significance values (p-values) derived from Linear (LM) and Generalized Linear Models (GLM).

| Species | Model (family; link) | Formula (structure) | AIC | p-values (Type II) |
| --- | --- | --- | --- | --- |
| AS | LM (Gaussian; identity) | GSH ~ Season | 30.067 | Season = 0.145 |
| CS | LM (Gaussian; identity) | GSH ~ Site * Season | −12.842 | Site = 0.213; Season < 0.001; Site×Season = 0.015 |
| GM | GLM (Gamma; log) | GSH ~ Season | 48.307 | Season < 0.001 |
| MN | GLM (Gamma; log) | GSH ~ Site | −9.917 | Site = 0.082 |
|  |  |  |  |  |

Table S9. Model-predicted reduced glutathione (GSH) levels in leaves of *Alchornea sidifolia* (AS), *Casearia sylvestris* (CS), *Guarea macrophylla* (GM), and *Machaerium nyctitans* (MN) across site × season combinations. Values are fitted means derived from the best-supported model selected separately for each species (Table S8). RMG, Morro Grande Forest Reserve; Matão-IAG, Biosciences Institute Forest Reserve. Values are expressed as µmol g⁻¹ fresh mass (FM).

| Species | RMG Dry | Matão-IAG Dry | RMG Rainy | Matão-IAG Rainy |  |
| --- | --- | --- | --- | --- | --- |
| AS | 1.58 | 1.58 | 1.33 | 1.33 | |
| CS | 0.542 | 0.616 | 0.47 | 0.212 | |
| GM | 2.21 | 2.21 | 0.48 | 0.48 | |
| MN | 0.439 | 0.27 | 0.439 | 0.27 | |

Table S10. Influence of site and season on oxidized glutathione (GSSG) levels in *Alchornea sidifolia* (AS), *Casearia sylvestris* (CS), *Guarea macrophylla* (GM), and *Machaerium nyctitans* (MN). Values indicate the Akaike Information Criterion (AIC) and significance values (p-values) derived from Linear (LM) and Generalized Linear Models (GLM).

| Species | Model (family; link) | Formula (structure) | AIC | p-values |
| --- | --- | --- | --- | --- |
| AS | GLM (Gamma; log) | GSSG ~ Site + Season | −80.483 | Site = 0.150; Season = 0.003 |
| CS | GLM (Gamma; log) | GSSG ~ Site * Season | −134.800 | Site = 0.243; Season < 0.001; Site×Season = 0.002 |
| GM | GLM (Gamma; log) | GSSG ~ Site + Season | −41.151 | Site = 0.087; Season = 0.023 |
| MN | LM (Gaussian; identity) | GSSG ~ Site * Season | −52.181 | Site = 0.146; Season = 0.082; Site×Season = 0.001 |

Table S11. Model-predicted oxidized glutathione (GSSG) levels in leaves of *Alchornea sidifolia* (AS), *Casearia sylvestris* (CS), *Guarea macrophylla* (GM), and *Machaerium nyctitans* (MN) across site × season combinations. Values are fitted means derived from the best-supported model selected separately for each species (Table S10). RMG, Morro Grande Forest Reserve; Matão-IAG, Biosciences Institute Forest Reserve. Values are expressed as µmol g⁻¹ fresh mass (FM).

| Species | RMG Dry | Matão-IAG Dry | RMG Rainy | Matão-IAG Rainy |  |
| --- | --- | --- | --- | --- | --- |
| AS | 0.136 | 0.096 | 0.066 | 0.046 | |
| CS | 0.027 | 0.04 | 0.014 | 0.004 | |
| GM | 0.262 | 0.14 | 0.112 | 0.06 | |
| MN | 0.063 | 0.195 | 0.103 | 0.045 | |

Table S12. Influence of site and season on total glutathione (TotalGSH) levels in *Alchornea sidifolia* (AS), *Casearia sylvestris* (CS), *Guarea macrophylla* (GM), and *Machaerium nyctitans* (MN). Values indicate the Akaike Information Criterion (AIC) and significance values (p-values) derived from Linear (LM) and Generalized Linear Models (GLM).

| Species | Model (family; link) | Formula (structure) | AIC | p-values |
| --- | --- | --- | --- | --- |
| AS | LM (Gaussian; identity) | TotalGSH ~ Season | 30.808 | Season = 0.078 |
| CS | GLM (Gamma; log) | TotalGSH ~ Site * Season | −17.817 | Site = 0.019; Season < 0.001; Site×Season < 0.001 |
| GM | GLM (Gamma; log) | TotalGSH ~ Season | 51.456 | Season < 0.001 |
| MN | GLM (Gamma; log) | TotalGSH ~ Site * Season | −1.410 | Site = 0.611; Season = 0.726; Site×Season = 0.004 |

Table S13. Model-predicted total glutathione (TotalGSH) levels in leaves of *Alchornea sidifolia* (AS), *Casearia sylvestris* (CS), *Guarea macrophylla* (GM), and *Machaerium nyctitans* (MN) across site × season combinations. Values are fitted means derived from the best-supported model selected separately for each species (Table S12). RMG, Morro Grande Forest Reserve; Matão-IAG, Biosciences Institute Forest Reserve. Values are expressed as µmol g⁻¹ fresh mass (FM).

| Species | RMG Dry | Matão-IAG Dry | RMG Rainy | Matão-IAG Rainy |  |
| --- | --- | --- | --- | --- | --- |
| AS | 1.69 | 1.69 | 1.39 | 1.39 | |
| CS | 0.569 | 0.655 | 0.501 | 0.216 | |
| GM | 2.4 | 2.4 | 0.56 | 0.56 | |
| MN | 0.378 | 0.598 | 0.619 | 0.288 | |

Table S14. Influence of site and season on glutathione half-cell reduction potential (E_hc_) in *Alchornea sidifolia* (AS), *Casearia sylvestris* (CS), *Guarea macrophylla* (GM), and *Machaerium nyctitans* (MN). Values indicate the Akaike Information Criterion (AIC) and significance values (p-values) derived from Linear (LM) and Generalized Linear Models (GLM).

| Species | Model (family; link) | Formula (structure) | AIC | p-values |
| --- | --- | --- | --- | --- |
| AS | LM (Gaussian; identity) | E_hc_ ~ Season | 185.380 | Season = 0.152 |
| CS | LM (Gaussian; identity) | E_hc_ ~ 1 (null) | 193.880 | — |
| GM | LM (Gaussian; identity) | E_hc_ ~ Season | 188.410 | Season = 0.059 |
| MN | LM (Gaussian; identity) | E_hc_ ~ 1 (null) | 208.920 | — |

Table S15. Influence of site and season on total phenolic content (TPC) in *Alchornea sidifolia* (AS), *Casearia sylvestris* (CS), *Guarea macrophylla* (GM), and *Machaerium nyctitans* (MN). Values indicate the Akaike Information Criterion (AIC) and significance values (p-values) derived from Linear (LM) and Generalized Linear Models (GLM).

| Species | Model (family; link) | Formula (structure) | AIC | p-values |
| --- | --- | --- | --- | --- |
| AS | LM (Gaussian; identity) | TPC ~ Site | 166.39 | Site = 0.001 |
| CS | GLM (Gamma; log) | TPC ~ Site + Season | 99.894 | Site = 0.010; Season = 0.143 |
| GM | LM (Gaussian; identity) | TPC ~ 1 (null) | 66.59 | — |
| MN | LM (Gaussian; identity) | TPC ~ Site + Season | 118.21 | Site = 0.106; Season = 0.019 |

Table S16. Influence of site and season on superoxide dismutase (SOD) activity in *Alchornea sidifolia* (AS), *Casearia sylvestris* (CS), *Guarea macrophylla* (GM), and *Machaerium nyctitans* (MN). Values indicate the Akaike Information Criterion (AIC) and significance values (p-values) derived from Linear (LM) and Generalized Linear Models (GLM).

| Species | Model (family; link) | Formula (structure) | AIC | p-values |
| --- | --- | --- | --- | --- |
| AS | LM (Gaussian; identity) | SOD ~ 1 (null) | 444.49 | — |
| CS | LM (Gaussian; identity) | SOD ~ 1 (null) | 381.67 | — |
| GM | LM (Gaussian; identity) | SOD ~ Season | 324.32 | Season = 0.096 |
| MN | GLM (Gamma; log) | SOD ~ Site | 372.48 | Site = 0.044 |

Table S17. Influence of site and season on catalase (CAT) activity in *Alchornea sidifolia* (AS), *Casearia sylvestris* (CS), *Guarea macrophylla* (GM), and *Machaerium nyctitans* (MN). Values indicate the Akaike Information Criterion (AIC) and significance values (p-values) derived from Linear (LM) and Generalized Linear Models (GLM).

| Species | Model (family; link) | Formula (structure) | AIC | p-values |
| --- | --- | --- | --- | --- |
| AS | LM (Gaussian; identity) | CAT ~ 1 (null) | 215.64 | — |
| CS | GLM (Gamma; log) | CAT ~ 1 (null) | 210.42 | — |
| GM | LM (Gaussian; identity) | CAT ~ Site * Season | 189.68 | Site = 0.502; Season = 0.042; Site×Season = 0.068 |
| MN | GLM (Gamma; log) | CAT ~ Site * Season | 227.13 | Site = 0.399; Season = 0.011; Site×Season < 0.001 |

Table S18. Influence of site and season on ascorbate peroxidase (APX) activity in *Alchornea sidifolia* (AS), *Casearia sylvestris* (CS), *Guarea macrophylla* (GM), and *Machaerium nyctitans* (MN). Values indicate the Akaike Information Criterion (AIC) and significance values (p-values) derived from Linear (LM) and Generalized Linear Models (GLM).

| Species | Model (family; link) | Formula (structure) | AIC | p-values |
| --- | --- | --- | --- | --- |
| AS | LM (Gaussian; identity) | APX ~ Site * Season | 387.47 | Site = 0.165; Season = 0.435; Site×Season = 0.026 |
| CS | GLM (Gamma; log) | APX ~ Season | 378.77 | Season = 0.006 |
| GM | LM (Gaussian; identity) | APX ~ 1 (null) | 360.82 | — |
| MN | GLM (Gamma; log) | APX ~ 1 (null) | 421.36 | — |

Table S19. Influence of site and season on malondialdehyde (MDA) levels in *Alchornea sidifolia* (AS), *Casearia sylvestris* (CS), *Guarea macrophylla* (GM), and *Machaerium nyctitans* (MN). Values indicate the Akaike Information Criterion (AIC) and significance values (p-values) derived from Linear (LM) and Generalized Linear Models (GLM).

| Species | Model (family; link) | Formula (structure) | AIC | p-values (Type II) |
| --- | --- | --- | --- | --- |
| AS | GLM (Gamma; log) | MDA ~ Season | 229.78 | Season < 0.001 |
| CS | LM (Gaussian; identity) | MDA ~ Season | 224.74 | Season < 0.001 |
| GM | LM (Gaussian; identity) | MDA ~ Season | 211.93 | Season = 0.103 |
| MN | GLM (Gamma; log) | MDA ~ Season | 229.91 | Season = 0.001 |

**Supplementary Methods**

**SM1. Non-enzymatic antioxidants: ascorbate (AsA) and glutathione (GSH)**

AsA and GSH pools, including their reduced forms (AsA and GSH), oxidized forms (dehydroascorbate (DHA) and glutathione disulfide (GSSG)), and total forms (AsA + DHA and GSH + GSSG), were quantified using a method adapted from Sala-Carvalho et al. (2022). Analyses were performed by high-performance liquid chromatography with diode-array detection (HPLC-DAD; LC1260, Agilent Technologies) using an Agilent Eclipse Plus C18 column (4.6 × 150 mm, 4.6 µm) maintained at 25 °C, a flow rate of 1 mL min⁻¹, and a 10 mm flow cell. The mobile phase consisted of water acidified with phosphoric acid (H₃PO₄, pH 2.3), and compounds were separated isocratically for 10 min. Detection was set at 245 nm for AsA and 194 nm for GSH.

For sample preparation, 150 mg of frozen ground leaf tissue was homogenized in 2 mL of 6% metaphosphoric acid (HPO₃) containing 0.5 M ethylenediaminetetraacetic acid (EDTA). After centrifugation at 10,000 rpm for 15 min at 4 °C, the supernatant was collected. To determine reduced AsA and GSH, 100 µL of supernatant was diluted with 400 µL of mobile phase, filtered through a polytetrafluoroethylene (PTFE) membrane (0.45 µm), and 50 µL was injected into the HPLC. To determine total AsA and total GSH, 100 µL of supernatant was mixed with 20 µL of 0.16% dithiothreitol (DTT) prepared in 2 M sodium phosphate buffer (pH 7.0) and 10 µL of 45% dipotassium hydrogen phosphate (K₂HPO₄), then incubated on dry ice in the dark for 20 min. The reaction was stopped with 20 µL of 0.2 M H₃PO₄. The mixture was diluted with 350 µL of water, filtered through a PTFE membrane (0.45 µm), and 50 µL was injected into the HPLC.

DHA and GSSG were obtained by subtracting the reduced forms from the corresponding total pools. All metabolite pools were expressed as µmol g⁻¹ FM.

**SM2. Total phenolic content (TPC)**

TPC was determined by the Folin–Ciocalteu method, following Furlan et al. (2015). Frozen ground leaf tissue (50 mg) was mixed with 1.5 mL of 80% methanol, sonicated for 15 min at room temperature, and centrifuged at 10,000 rpm for 10 min. An aliquot of 50 μL of supernatant was mixed with 190 μL of ultrapure water, 10 μL of Folin–Ciocalteu reagent, and 50 μL of 10% sodium carbonate (Na₂CO₃). After incubation at 40 °C for 30 min, absorbance was measured at 760 nm. Quantification was based on a gallic acid standard curve prepared in 80% methanol (5–80 μg mL⁻¹; R² = 0.99), and TPC was expressed as mg g⁻¹ FM.

**SM3. Antioxidant enzyme activities**

Enzyme extraction and activity assays followed Lopes & Furlan (2025). Frozen tissue (250 mg) was homogenized in 1.5 mL extraction buffer containing 50 mM potassium phosphate buffer (pH 7.8), 5 mM AsA, 2 mM EDTA, 12 mM DTT, and 30 mg polyvinylpolypyrrolidone (PVPP). After centrifugation at 10,000 rpm for 10 min at 4 °C, the supernatant was collected and stored at −80 °C.

CAT activity was measured in UV-transparent 96-well microplates containing 182 µL of 100 mM potassium phosphate buffer (pH 7.5), 8 µL of enzyme extract, and 10 µL of 200 mM hydrogen peroxide (H₂O₂). The decrease in absorbance at 240 nm was recorded at 25 °C every 30 s for 2 min. Activity was calculated from the rate of H₂O₂ decomposition using a molar extinction coefficient of 40 M⁻¹ cm⁻¹ (Noctor et al., 2016) and expressed as µmol H₂O₂ min⁻¹ g⁻¹ FM.

APX activity was determined from the decrease in absorbance at 290 nm associated with H₂O₂-dependent ascorbate oxidation. The assay mixture consisted of 150 µL of 100 mM potassium phosphate buffer (pH 7.0) containing 1 mM EDTA, 20 µL of 5 mM AsA, 20 µL of enzyme extract, and 10 µL of 200 mM H₂O₂. Absorbance was recorded every 30 s for 3 min. Enzyme activity was calculated using an extinction coefficient of 2.8 mM⁻¹ cm⁻¹ (Lopes & Furlan, 2025) and expressed as µmol AsA oxidized min⁻¹ g⁻¹ FM.

SOD activity was determined from inhibition of nitro blue tetrazolium (NBT) photoreduction. The reaction mixture contained 50 mM potassium phosphate buffer (pH 7.8), 0.1 mM EDTA, 0.4 mM NBT, 5 mM L-methionine S-oxide, and 0.014 mM riboflavin. Aliquots of 190 µL of reaction mixture and 10 µL of enzyme extract were combined in UV-transparent microplates and exposed to light for 20 min (80 W). A duplicate set was kept in the dark as a control. Absorbance was read at 560 nm. One unit of SOD activity was defined as the amount of enzyme causing 50% inhibition of NBT photoreduction (Lopes & Furlan, 2025) and expressed as U g⁻¹ FM.

**SM4. Malondialdehyde (MDA)**

MDA equivalents were determined using a thiobarbituric acid-reactive substances (TBARS) assay based on Hodges et al. (1999). Frozen ground leaf tissue (200 mg fresh mass, FM) was homogenized in 1.5 mL of 80% ethanol and kept on ice in the dark for 1 h. After centrifugation at 13,000 rpm for 10 min, 100 µL of the supernatant was mixed with 100 µL of a reaction solution containing 2% trichloroacetic acid (TCA), 0.1% ascorbic acid (AsA), and 0.65% thiobarbituric acid (TBA) in water. The mixture was vortexed for 30 s and heated at 90 °C for 30 min. The reaction was stopped by cooling on ice for 10 min. Samples were then transferred to a 96-well UV microplate, and absorbance was read at 440, 532, and 600 nm. For each sample, a negative control (−TBA) was prepared by mixing 100 µL of supernatant with a solution containing 2% TCA and 0.1% AsA and processing it under the same conditions.

Corrected absorbance was calculated as A − B, where:

A = [(Abs₅₃₂(+TBA) − Abs₆₀₀(+TBA)) − (Abs₅₃₂(−TBA) − Abs₆₀₀(−TBA))]

B = (Abs₄₄₀(+TBA) − Abs₆₀₀(+TBA)) × 0.0571

MDA equivalents (nmol mL⁻¹) were obtained from an MDA standard curve (2–16 nmol mL⁻¹; R² = 0.99), corrected for extraction volume, and normalized to fresh mass (nmol g⁻¹ FM).

**References**

Furlan, C. M., Santos, K. P., Sedano-Partida, M. D., Motta, L. B. da, Santos, D. Y. A. C. dos, Salatino, M. L. F., Negri, G., Berry, P. E., van Ee, B. W., & Salatino, A. (2015). Flavonoids and antioxidant potential of nine Argentinian species of *Croton* (Euphorbiaceae). *Brazilian Journal of Botany, 38*(4), 693–702. <https://doi.org/10.1007/s40415-014-0115-9>

Hodges, D. M., DeLong, J. M., Forney, C. F., & Prange, R. K. (1999). Improving the thiobarbituric acid-reactive-substances assay for estimating lipid peroxidation in plant tissues containing anthocyanin and other interfering compounds. *Planta, 207*, 604–611. <https://doi.org/10.1007/s004250050524>

Lopes, A. S., & Furlan, C. M. (2025). Seasonal regulation of the antioxidant systems confers resilience in the moss *Brittonodoxa subpinnata*. *Theoretical and Experimental Plant Physiology, 37*, Article 44. <https://doi.org/10.1007/s40626-025-00389-w>

Noctor, G., Mhamdi, A., & Foyer, C. H. (2016). Oxidative stress and antioxidative systems: Recipes for successful data collection and interpretation. *Plant, Cell & Environment, 39*(5), 1140–1160. <https://doi.org/10.1111/pce.12726>

Sala-Carvalho, W. R., Montessi-Amaral, F. P., Esposito, M. P., Campestrini, R., Rossi, M., Peralta, D. F., & Furlan, C. M. (2022). Metabolome of *Ceratodon purpureus* (Hedw.) Brid., a cosmopolitan moss: The influence of seasonality. *Planta, 255*, Article 77. <https://doi.org/10.1007/s00425-022-03857-8>
